# Phylogenetic analysis of the caspase family in bivalves: implications for programmed cell death, immune response and development

**DOI:** 10.1186/s12864-021-07380-0

**Published:** 2021-01-25

**Authors:** Susanne Vogeler, Stefano Carboni, Xiaoxu Li, Alyssa Joyce

**Affiliations:** 1 Department of Marine Science, University of Gothenburg, Carl Skottbergsgata 22 B, 41319 Gothenburg, Sweden; 2 Institute of Aquaculture, University of Stirling, Stirling, Scotland FK9 4LA UK; 3 South Australia Research and Development Institute Aquatic Sciences Centre, 2 Hamra Ave, West Beach, SA 5024 Australia

**Keywords:** Caspase, Apoptosis, Bivalves, Innate immune system, Programmed cell death, Inflammation response, Pyroptosis

## Abstract

**Background:**

Apoptosis is an important process for an organism’s innate immune system to respond to pathogens, while also allowing for cell differentiation and other essential life functions. Caspases are one of the key protease enzymes involved in the apoptotic process, however there is currently a very limited understanding of bivalve caspase diversity and function.

**Results:**

In this work, we investigated the presence of caspase homologues using a combination of bioinformatics and phylogenetic analyses. We blasted the *Crassostrea gigas* genome for caspase homologues and identified 35 potential homologues in the addition to the already cloned 23 bivalve caspases. As such, we present information about the phylogenetic relationship of all identified bivalve caspases in relation to their homology to well-established vertebrate and invertebrate caspases. Our results reveal unexpected novelty and complexity in the bivalve caspase family. Notably, we were unable to identify direct homologues to the initiator caspase-9, a key-caspase in the vertebrate apoptotic pathway, inflammatory caspases (caspase-1, − 4 or − 5) or executioner caspases-3, − 6, − 7. We also explored the fact that bivalves appear to possess several unique homologues to the initiator caspase groups − 2 and − 8. Large expansions of caspase-3 like homologues (caspase-3A-C), caspase-3/7 group and caspase-3/7-like homologues were also identified, suggesting unusual roles of caspases with direct implications for our understanding of immune response in relation to common bivalve diseases. Furthermore, we assessed the gene expression of two initiator (Cg2A, Cg8B) and four executioner caspases (Cg3A, Cg3B, Cg3C, Cg3/7) in *C. gigas* late-larval development and during metamorphosis, indicating that caspase expression varies across the different developmental stages.

**Conclusion:**

Our analysis provides the first overview of caspases across different bivalve species with essential new insights into caspase diversity, knowledge that can be used for further investigations into immune response to pathogens or regulation of developmental processes.

**Supplementary Information:**

The online version contains supplementary material available at 10.1186/s12864-021-07380-0.

## Background

**Fig. 1:**
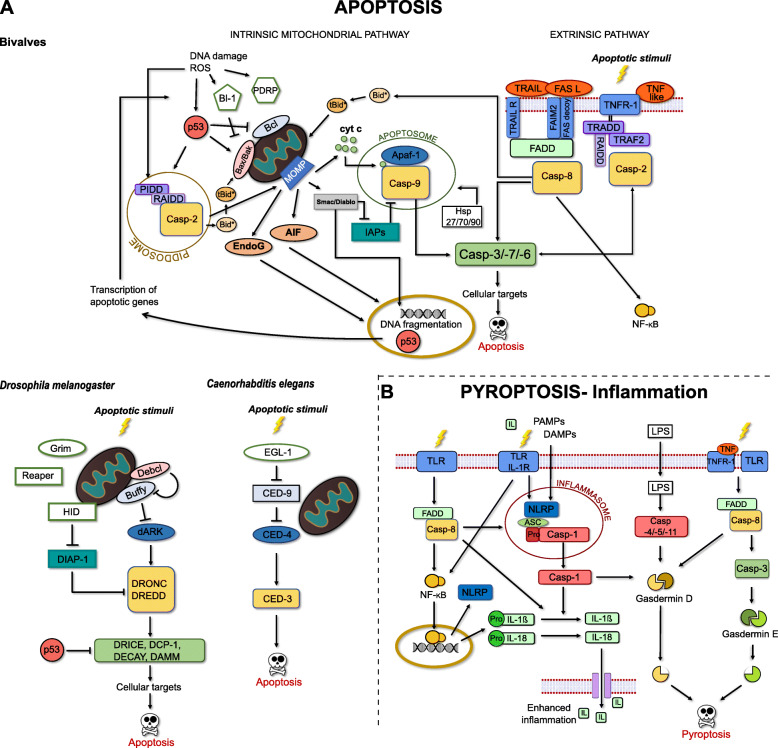
Schematic representation of **a** potential apoptotic pathways in bivalve species based on homologous genes characterised in bivalves or suggested in bivalve genomes (*not identified in a bivalve species yet) to the vertebrate’s intrinsic mitochondrial or extrinsic apoptotic pathways as well as apoptotic pathways in *Drosophila melanogaster* and *Caenorhabditis elegans*. **b** Pyroptotic pathways in vertebrates. (Adopted from [6–16])

Bivalves, with their aquatic life style and often limited mobility, have evolved a diverse repertoire of defence strategies to eliminate pathogens. The innate immune system of bivalves, including cellular and humoral responses, is one of the most important and sophisticated defence mechanisms among invertebrates for pathogen recognition and elimination [1, 2]. One of these strategies includes apoptosis, a type of programmed cell death, to prevent the spread of pathogens within the organism [3]. Apoptosis leads to cell death of infected or unwanted cells, with cell shrinkage and nuclear fragmentation followed by phagocytosis of the apoptotic bodies by neighbouring cells, without needing to elicit an inflammatory response. Pathogens on the other hand, are seeking tactics to prevent apoptosis, for instance by inhibiting catalytic enzymes, or through strategies that avoid triggering the host cell response. Apoptosis is also involved in key developmental processes for organ differentiation and formation of structures in vertebrates and invertebrates alike [4]. Apoptosis has been widely studied in molluscan species [3, 5, 6] and a comparison between apoptotic pathways of pre-bilaterian, ecdysozoan (insects & nematodes) and vertebrate models has revealed that the complex process of apoptosis in bivalve species shares many apoptosis-related genes with deuterostomes (Fig. 1a) [6, 7, 17]. By contrast, ecdysozoan apoptotic pathways such as in *Caenorhabditis elegans* and *Drosophila melanogaster* seem to be much simpler as a result of lineage specific gene losses.

### Caspase-dependent pathways in programmed cell death

Although apoptosis requires a diverse group of proteins, receptors and enzymes, the key component of apoptotic pathways are caspases: protease enzymes that initiate and execute all other processes [8]. Generally, caspases are differentiated into initiator caspases (caspase-2, − 8, − 9, − 10) and executioner caspases (caspase-3, − 6, − 7). Caspases are present in the cell as inactive zymogens containing a prodomain at the N-terminal and a large subunit (p20) followed by a small subunit (p10) towards the C-terminal. The prodomains of initiator caspases are often longer, containing homotypic interaction motifs such as the caspase-recruitment domain (CARD) in caspase-2 and caspase-9 and death-effector domains (DEDs) in caspase-8 and caspase-10 that function as recruitment domains. Caspases are cleaved by facilitating proteins to remove the prodomain and separate the large and small subunit at the intersubunit linker, which leads to the formation of a heterodimer of both subunits. To be activated, two heterodimers form a caspase dimer-complex [18, 19] with the catalytic histidine/cysteine dyad (active sites in p20 subunit) free to hydrolyse peptide bonds of target proteins [20, 21]. Two major apoptotic pathways exist in deuterostomes and are similarly proposed for molluscan species: the extrinsic and intrinsic pathway (Fig. 1a) [8, 22]. The extrinsic pathway is activated by receiving apoptotic signals at the cell surface by transmembrane receptors, which then trigger the auto-catalytic activation of the initiator caspases-8. Activated caspases-8 cleave and activate the executioner caspases-3, − 7 or − 6, which regulate the final apoptotic events such as DNA fragmentation, plasma blebbing and proteolysis of key structural and cell cycle proteins including activation of additional executioner caspases [23]. The intrinsic mitochondrial pathway is a non-receptor-mediated pathway with stimuli coming from various sources, for instance UV radiation, reactive oxygen species (ROS), mitochondrial DNA damage, viral infection and environmental pollutants [8, 22]. In the centre of this proposed pathway are caspases-9, which form apoptosomes with apoptotic protease activating factor-1 (Apaf-1) and cytochrome c (Cyt c), and are regulated by various proteins associated with the mitochondria or in the cytoplasm. Caspase-2 is another initiator caspase, which potentially takes part in both apoptotic pathways as part of a PIDDosome or it can be activated via transmembrane tumour necrosis factor (TNF) receptor-related signals, but its actual pathways in the apoptotic process remain controversial [9].

Apart from apoptosis, caspases are also involved in an additional non-apoptotic cell death type, called pyroptosis, which is often linked to inflammatory response [10, 24]. This pathway, mostly described for vertebrates (Fig. 1b), uses its own pro-inflammatory caspases (caspase-1, − 4, − 5, − 11) usually including a CARD prodomain. These and other caspases trigger an inflammatory response mainly via cleaving interleukins (e.g. IL-1β or IL-18), cytokines important in cell signalling, or gasdermins, effector molecules which catalyse pyroptosis [11]. The key caspase for vertebrate inflammation is caspase-1, which gets activated after signals from pathogen-associated molecular patterns (PAMPs) or a host-cell generated danger-associated molecular patterns (DAMPs) are received, leading to the formation of an inflammasome with the procaspase-1 and associated proteins via their CARD-domains.

### Caspases in bivalves: an incomplete story

Besides being involved in the immune response, caspases also take part in developmental processes, including embryonal development in animals and humans, as well as cell differentiation, proliferation, learning and dendric pruning among other functions [4]. Several caspases have been identified in bivalve species with homologues to caspase-8 [25–29], caspase-2 [12, 26, 30], caspase-1 [12, 31], caspase-3 [30, 32–35], caspase-6 [35] and a potential bivalve specific group of caspase-3/7 [26, 36]. Most of these bivalve caspases were assumed to be involved in apoptotic processes in relation to haemocytes responses to pathogen infections [25, 26, 29, 31, 32, 34, 37–39], environmental stressors [26, 28, 35, 36, 39] or developmental processes [30, 33]. Nevertheless, apoptotic pathways and caspase functions are far from being well understood in bivalve species, with many essential caspases and pathways not identified or characterised; for instance, no functional caspase-9 homologue has been characterised to-date, even though this caspase is central to all other apoptotic pathways. Indeed, the rise in whole genomes and transcriptomes available for various bivalve species has helped our understanding of the presence and functions of caspases. Unfortunately, genes and transcripts are mostly annotated automatically, and naming of bivalve genes are based on their closest vertebrate homologues without further phylogenetic or functional analysis to confirm their accurate classification. This could lead to inaccurate assumptions that bivalve pathways function similarly to vertebrate systems. Moreover, various discrepancies in caspase classification appear to have occurred in previous bivalve studies. Cloned Pacific oyster *Crassostrea gigas* caspase-1 [12, 31] displays an identical protein sequence to another cloned *C. gigas* caspase-3 [32], while an additional caspase-3 homologue [33] differs from the prior mentioned caspase-3. Further caspase-3/7 homologues in *C. gigas* [36], and the mussel *Mytilus galloprovincialis* [26] also suggest a bivalve-specific caspase group, thus indicating a much more complex caspase family present in bivalves than previously suggested.

To examine the caspase family in bivalves, we investigated the presence of caspase homologues using a combination of bioinformatics and phylogenetic analyses. We blasted the *C. gigas* genome for caspase homologues and identified 35 potential homologues in the addition to the already cloned caspases in bivalves. Phylogenetic analyses of these bivalve caspases, as compared to homologues in other invertebrates and vertebrate species, confirmed expansions of the initiator and executioner caspase groups while also suggesting a need to correct some of the identifications of previously classified caspases. The identified homologues are discussed in relation to their potential implications for apoptosis, immune response and during development. The previously identified *C. gigas* caspases, and an additional potential caspase-3 homologue, were also used in an expression study in Pacific oyster larvae prior and after initiation of metamorphosis with the neurotransmitter epinephrine. Given that caspases are involved in such a wide variety of essential pathways, this analysis of caspases in bivalves brings new insight to their potential function, as well as correcting potentially misleading information from previous classification attempts. As such, we provide a solid foundation from which new directions can emerge that further our understanding of immune responses and developmental processes in bivalves.

## Results

### Phylogenetic assessment caspases in bivalves

Thirty-five putative caspases have been identified in the Pacific oyster genome in addition to the 23 caspase homologues previously characterised in bivalve species [12, 25–36]. Of these 35 putative caspases, twenty-seven have already been identified as caspase homologues by the automated annotation process during the genome assembly, although only 16 have been classified in similar caspase groups as presented in the phylogenetic analysis of this study (Additional file 1). All identified bivalve caspases possess a large p20 caspase subunit unique for caspase homologues. However, 10 of the identified *C. gigas* caspase homologues in the oyster genome only contain a p20 subunit without a downstream small p10 subunit based on a conserved motif search with ScanProsite. A re-blast of these caspases to vertebrates, non-vertebrate metazoans or non-metazoan of the NCBI protein database showed high homologies to the metazoans characterised, or proposed caspases with no significant homology to other protein groups. Of all 46 bivalve caspases with p20 and p10 subunits, nine bivalve caspase homologues contain CARDs in their prodomains, five have two DEDs in the prodomains, two homologues have an additional death domain (DD) motif after the two DEDs, four homologues have only one DED domain, as well as two homologues that have two caspase-unusual domains in their prodomain, the double stranded RNA-binding domains (DSRM). The remaining 24 caspases are relatively short without any specific domain in their prodomains. Trimmed CASc domains (caspase-specific domains, p20 and p10 subunits without intersubunit linker) of CARD or DED domains-possessing caspase homologues were aligned with known initiator caspases-2, − 9, − 8 and − 10 of other species, as well as vertebrate inflammatory caspases-1, − 4, and − 5, which also contain CARDs. The remaining bivalve caspase homologues were aligned with known executioner caspase-3, − 6 and − 7 homologues.

**Fig. 2:**
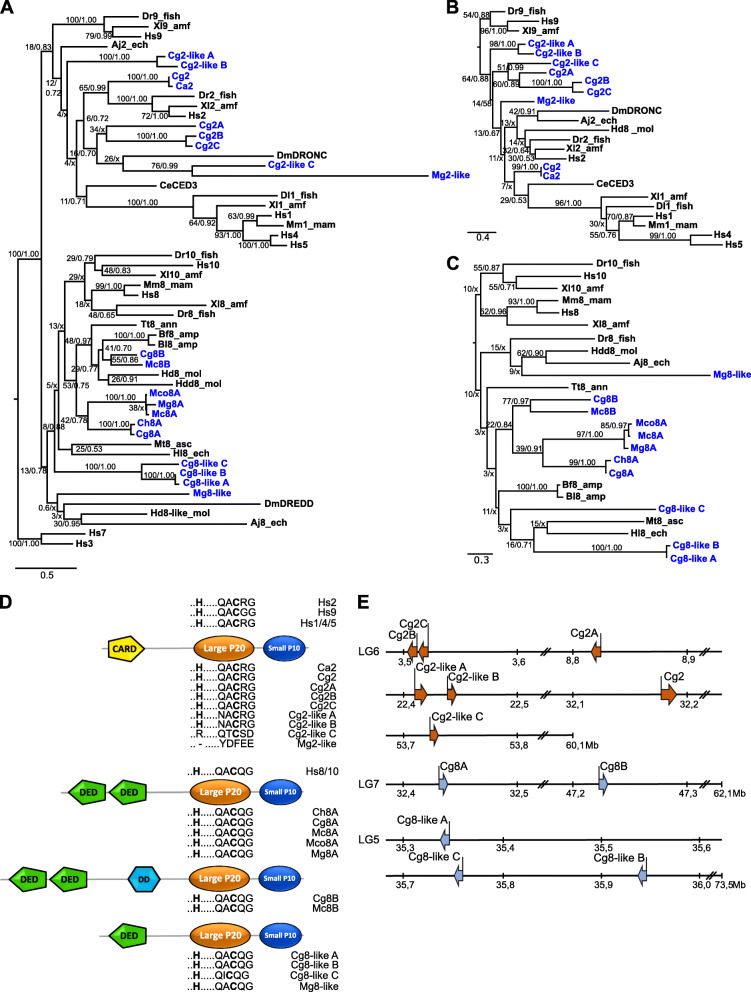
**a** Phylogenetic relationship of initiator caspases in bivalves (blue) compared to other vertebrate and invertebrate homologues (black). Values above/below nodes separated by slash show bootstrap support values for Maximum Likelihood (ML) analysis as percentage of bootstrap values for the main tree with additional Bayesian Inference (BI) posterior probabilities. /x indicates the nodes obtained from the BI which were different from the ML analysis. Human caspase-3 and caspase-7 homologues used as outgroup. Phylogenetic relationship of caspase-recruitment domain (CARD) **b** or single/double death-effector domains (DED) **c**. **d** Schematic representation of initiator caspase structure of bivalves with the CARD, DED or death domain (DD) motifs in their prodomains and the two caspase specific domains: large p20 and small p10 domain. The p20 active sites motif (..H … ..QACXG) with the conserved histidine and cysteine residue in bold is shown for each bivalve caspase homolog. **e** Schematic representation of gene location for each identified *C. gigas* caspase on the pseudo-chromosomes (LG). Mb: megabase. Aj: *Apostichopus japonicus*, Bf: *Branchiostoma floridae,* Bl: *Branchiostoma lanceolatum*, Ca: *Crassostrea angulata*, Ce: *Caenorhabditis elegans*, Cg: *Crassostrea gigas*, Ch: *Crassostrea hongkongensis*, Dl: *Dicentrarchus labrax*, Dm: *Drosophila melanogaster*, Dr.: *Danio rerio*, Hd: *Haliotis diversicolor,* Hdd: *Haliotis discus discus,* Hl: *Holothuria leucospilota*, Hs: *Homo sapiens*, Mc: *Mytilus californianus,* Mco: *Mytilus coruscus,* Mg: *Mytilus galloprovincialis,* Mm: *Mus musculus,* Mt: *Molgula tectiformis,* Tt: *Tubifex tubifex,* Xl: *Xenopus laevis.* _amf: amphibian, −amp: amphioxus, _ann: annelid, _asc: ascidian, _ech: echinoderm, _fish: fish, _mam: mammal, _mol: mollusc

The phylogenetic analysis of the CASc domains of initiator caspases included two clades, with one group including all CARD-containing caspases for caspase-2, caspase-9 and inflammatory caspases, and a second group that included DED-containing caspase-8 and caspase-10 homologues (Fig. 2a). In general, the initiator caspase divergence from the executioner caspases (outgroup Hs3 and Hs7) was highly supported in both phylogenetic analyses (Maximum Likelihood (ML) bootstrap percentage: 100%; Bayesian inferences (BI) posterior probabilities: 1.00). The CASc domains of CARD-containing initiator caspases revealed that the nine bivalve CARD-containing caspases showed the highest homology to caspase-2 homologues with no direct homologue found to vertebrate caspase-9 or to the inflammatory caspase group. This classification was also supported by a separate phylogenetic analysis of the CARD domains (Fig. 2b), of which none directly grouped with either of the vertebrae caspase-9 or inflammatory caspase CARD clades. Intron/exon assessment of the CARD domains of the oyster caspases have revealed a similar composition to vertebrate CARD domains of caspase-2 and caspase-9 homologues, with each domain encoded by two exons. CARD domains of vertebrate caspase-1, − 4 and − 5, on the other hand, are encoded by one exon. Furthermore, the p20 active site motifs of Ca2, Cg2, Cg2A (previously identified as Cg2 [12]), Cg2B and Cg2C were identical to human caspase-2 homologue Hs2 with a QACRG motif and not to the human caspase-9 Hs9 motif QACGG (Fig. 2d). However, a QACRG motif is also present in inflammatory caspases, but based on the position of these bivalve caspases in the phylogenetic tree, it is less likely that these caspases were homologues to the inflammatory caspase, although similar functional characteristics cannot be excluded. The remaining bivalve caspase-2-like homologues displayed very different p20 motifs, although the three Cg2-like homologues contained the conserved cysteine in this motif. However, in contrast to the other initiator caspases, Cg2-like C contained an arginine instead of the conserved histidine residue ahead of the p20 motif QARXG. An outlier to all proposed bivalve caspase-2 homologues was the *M. galloprovincialis* caspase-2 homologue Mg2-like (previously identified as Mg2 [26]), which neither containing the conserved cysteine or histidine residue. Based on the most recent assembly of the oyster genome, which has assembled the genome into 10 pseudo-chromosomes (linkage groups LGs) [40], all caspase-2 gene homologues are located on pseudo-chromosomes LG6, mostly separated by several megabases except for Cg2B and Cg2C as well as Cg2-like A and Cg2-like B (Fig. 2e). These caspase-2 homologues are closely located into two groups, suggesting *C. gigas* specific gene duplications.

The second clade containing caspase-8 and caspase-10 homologues possessed 11 of the identified bivalve caspases of which three (Cg8-like A-C) were newly identified in the *C. gigas* genome (Fig. 2a). Rather than being direct homologues to either vertebrate caspase-8 or caspase-10 members, they grouped outside the vertebrate caspase-8/10 group in three small groups: caspase-8A, caspase-8B and a caspase-8-like group. The bivalve caspase-8A group was clustered together according to their species genus based on the three *Mytilus* caspases-8A and the two *Crassostrea* caspases-8A. The two bivalve caspase-8B homologues, Cg8B (previously described as Cg8 [25]) and Mc8B, containing two DEDs and a DD domain in their prodomains, also grouped together based on their CASc domain sequence. Similar phylogenetic arrangements were seen for the DED domain analysis (Fig. 2c). The four bivalve caspase-like homologues, Cg8-like A-C and Mg8-like (previously identified as Mg8 [26]), however, were less conserved in direct comparison to the CASc and prodomain phylogenetic positions, and only contained one DED each. Nevertheless, with one DED in their prodomains, and the conserved caspase-8 p20 motif QARQG (except for Cg8-like C motif QICQG) present (Fig. 2d), supported by their position in the phylogenetic tree, these four bivalve caspases are likely homologues of caspases-8/10. Sequence analysis of each oyster caspase further has shown that each DED and DD domain is encoded by a single exon for each domain. Moreover, while Cg8A and Cg8B are located several megabases apart on pseudo-chromosome LG7, the Cg8-like group are located closer together on pseudo-chromosome LG5.

**Fig. 3:**
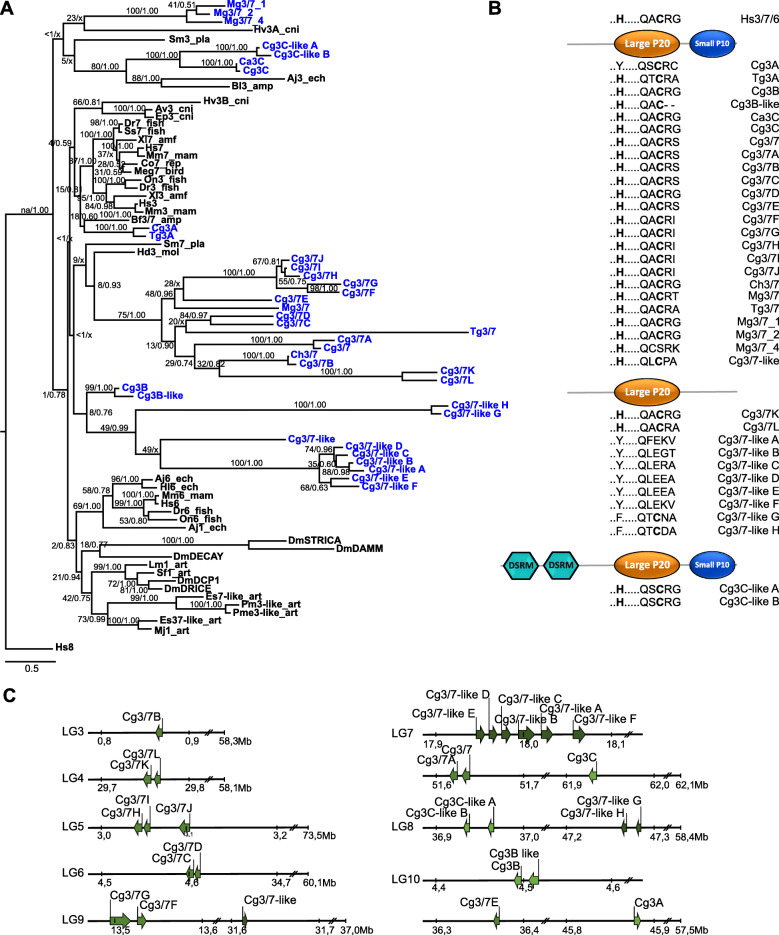
**a** Phylogenetic relationship of executioner caspases in bivalves (blue) compared to other vertebrate and invertebrate homologues (black). Values above/below branches separated by slash show bootstrap support values for Maximum Likelihood (ML) analysis as percentage of bootstrap values for the main tree with additional Bayesian Inference (BI) posterior probabilities. /x indicates the nodes obtained from the BI which were different from the ML analysis. Human caspase-8 used as outgroup. **b** Schematic representation of caspase structure with two caspase specific domains: large p20 and small p10 domain. The p20 active sites motif (..H … ..QACXG) with the conserved histidine and cysteine residue in bold is shown for each bivalve caspase homolog. DSRM: double stranded RNA-binding motif. **c** Schematic representation of gene location for each identified *C. gigas* caspase on the pseudo-chromosomes (LG). Mb: megabase pairs. Aj: *Apostichopus japonicus*, Av: *Anemonia viridis*, Bl: *Branchiostoma lanceolatum*, Bf: *Branchiostoma floridae,* Ca: *Crassostrea angulata*, Cg: *Crassostrea gigas*, Ch: *Crassostrea hongkongensis,* Co: *Cynops orientalis*, Dm: *Drosophila melanogaster*, Dr.: *Danio rerio*, Ep: *Exaiptasia pallida*, Es: *Eriocheir sinensis,* Hd: *Haliotis diversicolor,* Hl: *Holothuria leucospilota*, Hs: *Homo sapiens*, Hv: *Hydra vulgaris*, Lm: *Locusta migratoria*, Meg: *Meleagris gallopavo,* Mg: *Mytilus galloprovincialis*, Mj: *Marsupenaeus japonicas*, Mm: *Mus musculus,* On: *Oreochromis niloticu,* Pm: *Penaeus monodon*, Pme: *Penaeus merguiensis* Sf: *Spodoptera frugiperda,* Sm: *Schistosoma mansoni,* Ss: *Salmo salar,* Tg: *Tegillarca granosa,* Xl: *Xenopus laevis.* _amf: amphibian, −amp: amphioxus, _art: arthropod, _bird: bird, _cni: cnidarian, _ech: echinoderm, _fish: fish, _mam: mammal, _mol: mollusc, _pla: plathelminth, _rep: reptile

The phylogenetic analysis of the executioner caspases CASc domains has shown a more complex relationship within this type of caspase, as well as more variety in the p20 active sites QXCXG (Fig. 3). Although highly supported by BI analysis (posterior probabilities: 0.78) as a clustering group to the outgroup Hs8 and generally highly supported in terms of direct homologues within the executioner caspase clade, positionings of the larger subclades were generally poorly supported by both analyses (Fig. 3a) and resulted in polytomy in the BI analysis. Thus, positioning of these subclades might change, when new information on additional executioner caspases emerges in the future. Nevertheless, the phylogenetic analysis of the potential bivalve executioner caspases revealed distinct clustering of the 36 bivalve caspases, with some clades potentially unique to bivalves. None of the bivalve caspases have shown a direct homology to either of the vertebrate groups (caspase-3, caspase-7 or caspase-6) or the clade of arthropod caspases. The two bivalve caspases, Cg3A and Tg3A (previously described as Tg3 [35]) grouped outside the vertebrate caspase-3 and caspase-7 clade. Interestingly, although Cg3A and Tg3A seem to be the closest homologues to vertebrate caspases-3/− 7, both p20 active sites varied in their amino acid sequence (Cg3A: QSCRG, Tg3A: QTCRA) compared to the commonly found vertebrate QACRG sequence (Fig. 3b). Moreover, the histidine residue in Cg3A was not conserved and contained a tyrosine at this position instead. Alignments of the full coding sequence (CDS) and protein sequences of the previously cloned *C. gigas* caspase homologues caspase-3 [32], caspase-1 [12], and caspase-1 [31] have shown that these caspase homologues are indeed nearly identical (Additional file 2) and appear to be different isoforms of the same caspase. For our phylogenetic analysis, the isoform identified in the *C. gigas* genomes was considered to be representative of this caspase (CASc domains 100% identical) and hereafter named Cg3B based on its position in the tree (Fig. 3a) and its active p20 sites and conserved residue (..H … QACRG), which is identical to the active sites in human executioner caspases. Cg3B did not show high homology to the inflammatory caspase group caspase-1 or the executioner arthropod caspase clade including arthropod caspases-1. A second Cg3B-like caspase was identified with moderate CASc sequence identity to Cg3B (74% identity), which is likely to be a result of a gene duplication event based on the close location of Cg3B and Cg3B-like genes on the pseudo-chromosome LG10 (Fig. 3c). However, a deletion event has occurred in Cg3B-like leading to the loss of the arginine and glycine residues in the p20 active site QACRG (Fig. 3b). Cg3C and Ca3C (previously identified as Cg3 [33] and Ca3 [30]) were grouping together with two additional novel *C. gigas* caspases Cg3C-like A and Cg3C-like B. Both Cg3C-like caspases contained the unique two DSRM domains in their prodomains and their genes are closely located on the pseudo-chromosome LG8 (Fig. 3c), suggesting further gene duplication events in the Pacific oyster genome. The bivalve caspase-3C group are homologues to the previously identified deuterostome caspases Aj3 (echinoderm *Apostichopus japonicus* [41]) and Bl3 (amphioxus *Branchiostoma lanceolatum* [42]).

Three subclades of caspase-3-like bivalve caspases were also identified. The largest group contained caspase-3/7 homologues, clustering further outside the vertebrate caspase-3 and caspase-7 groups, the bivalve caspase-3A group and the cnidarian caspase-3 homologues, Av3 [43] and Ep3 [44] (Fig. 3a). This group contained members of several bivalve species with Ch3/7, Tg3/7, Mg3/7 and Cg3/7 (all previously identified as Ch3 [34], Tg6 [35], Mg3/7_3 [26] and Cg3/7 [36]) as well as 11 newly identified *C. gigas* caspases Cg3/7A-L. Conserved residues and active sites in the p20 subunits are mostly preserved with ..H … ..QACRX only varying in the last residue of the active site. Cg3/7 K and Cg3/7 L did not contain a p10 subunit (Fig. 3b). Interestingly, gene locations of all Cg3/7 caspases are dispersed across several pseudo-chromosomes and are only partly clustered together closely on the same pseudo-chromosome (Fig. 3c), with mostly two Cg3/7 homologues clustered together with few exceptions: Cg3/7-H, −I and -J on LG5, and two single caspase genes with Cg3/7B on LG3 and Cg3/7E on LG10. Closely located genes corresponded to close phylogenetic relationship of the Cg3/7 group.

A second large group named caspase-3/7-like (it positions even further from vertebrate caspase-3 and caspase-7) only comprised newly identified *C. gigas* caspases, with most of them containing only a p20 subunit and highly diverse p20 active sites (Fig. 3a & 3b). Gene location analysis revealed that Cg3/7-like A-F are closely clustered together on pseudo-chromosome LG7, indicating several gene duplication events of this caspase sub-group (Fig. 3c). Cg3/7-like H and Cg3/7-like G are clustered together on pseudo-chromosome LG8. The Cg3/7-like, the only member of this group that contained the conserved histidine in the p20 subunit however, is located separately on pseudo-chromosome LG9.

The third group combined three previously identified Mg3/7 homologues, for which previous phylogenetic analysis already showed unique clustering within executioner caspases together with an caspase-3 homologue in *Hydra vulgaris* Hv3A [26, 45]. However, other bivalve homologues to this group were not identified in the current analysis, and thus naming of these mussel caspase homologues remain as previously reported (Fig. 3a).

### Gene expression: caspases during larval development and metamorphosis

**Fig. 4:**
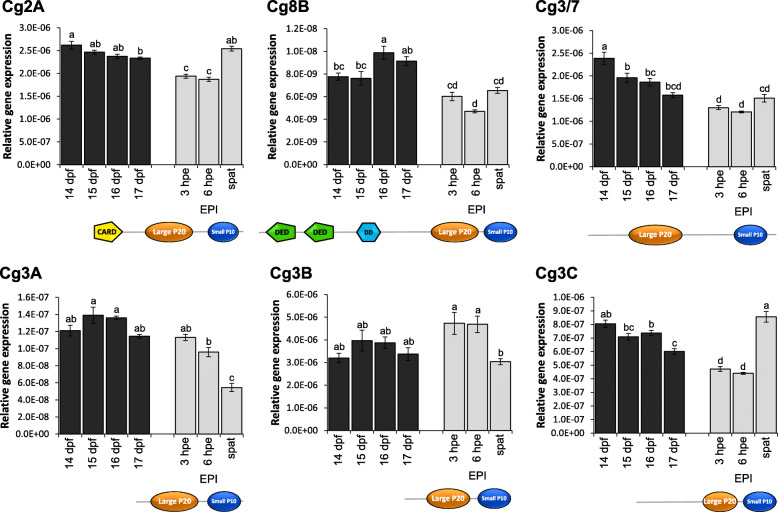
Relative gene expression of two initiator caspases, Cg2A and Cg8B, and four executioner caspases, Cg3/7, Cg3A, Cg3B, Cg3C, during Pacific oyster late larval development (14–17 days post fertilisation (dpf)), after 3 and 6 h post exposure start (3 hpe & 6 hpe) to a 3 h epinephrine (EPI) exposure at 10^− 4^ M for metamorphosis induction, and in spat (24 hpe). Each gene expression profile is accompanied by a schematic representation of the key domains present in the protein sequence of the analysed caspase. Different lower-case letters represent significant differences with *p* < 0.05. CARD: caspase-recruitment domain, DED: death-effector domain, DD: death domain, Large p20: large caspase subunit p20, Small p10: small caspase subunit p10

The gene expression profile of the five previously cloned Pacific oyster *C. gigas* caspases, Cg2A [12], Cg8B [25], Cg3/7 [36], Cg3B [12, 31, 32] and Cg3C [33], as well as the best hit for a caspase-3 homologue in our BLAST search of the *C. gigas* genome, Cg3A, were assessed during late larval development, after induction of metamorphosis and in spat to investigate if these caspases are potentially involved in the metamorphotic transition from larvae to spat in the Pacific oyster. Induction of metamorphosis in competent oyster larvae was successfully achieved after exposure to the well-known metamorphosis inducer epinephrine [46] for 3 h with epinephrine resulting in 81.3 ± 2.2% metamorphosis compared to 3.1 ± 0.3% metamorphosis in non-treated animals (*p* < 0.01). All caspase genes were expressed in all larvae and spat sample points displaying unique expression profiles (Fig. 4). The expression of initiator caspase Cg2A significantly decreased in larvae 14 days post fertilisation (dpf) to 17 dpf, a time during which larvae reached competence for metamorphosis, and again after 3 h post exposure start (hpe) and 6 hpe to epinephrine. In spat (24 hpe), Cg2A expression significantly increased again to a level comparable to that observed prior to induction of metamorphosis. The second proposed initiator caspase, Cg8B, on the other hand, increased its expression just prior to competence in 16 dpf, but expression then decreased during and post metamorphosis. The four executioner caspases also displayed slight differences in their expression profiles, with Cg3/7 expression significantly decreased throughout larval development and after metamorphosis induction, and remained low in spat. Cg3A expression was steady during development, but decreased after 6 hpe with a significant decrease observed in spat. Cg3B showed a similar profile as Cg3A, but expression increased slightly during metamorphosis, followed by a significant decrease in expression for spat. A decrease in expression throughout development and metamorphosis was also seen for Cg3C, but contrary to the other executioner caspases, Cg3C expression increased again in spat.

## Discussion

### Caspases in bivalves and immune system function

Caspases are key players in the apoptotic and inflammation processes in most vertebrates and invertebrates, hence the diversity of caspases present in bivalve species is not surprising. The phylogenetic relationship of the 56 bivalve caspases shows that bivalves possess caspase homologues that are different from the well-known vertebrate caspase groups, with expansions in both the initiator and executioner caspase groups. Interestingly, *C. gigas* and other bivalves contain several caspases where the histidine and cysteine residues in the p20 subunit were not conserved. These bivalve caspases may have lost their catalytic function, given that histidine and cysteine are essential for this process [21]. However, other amino acids besides these conserved regions are also responsible for substrate specificity, and detailed analysis of each caspase homologue is required to fully understand their function. Furthermore, some of the newly identified *C. gigas* caspases do not contain a p10 subunit; p10-minus caspase like structures have been previously reported in non-metazoan species as metacaspase-like proteins, with metacaspases being caspase homologues present in prokaryotes up to the level of higher plants [47, 48]. However, a re-BLAST of these unusual bivalve caspases has shown only homologies to other metazoan caspases, supporting the assumption that these are caspase-like proteins. While the metacaspase-like proteins of non-metazoan are suggested to be non-functional, or potentially vary in their substrate specificity to traditional caspases [48], further research is needed to clarify whether these unusual bivalve caspases are functional specifically in combination with histidine/cysteine residue mutations.

The specific bivalves studied here, possess a variety of initiator caspases, with either CARD or DED motifs present in their prodomains. However, the absence of a direct vertebrate caspase-9 homologue is particularly notable. None of the identified bivalve caspases possessed the caspase-9 typical p20 active site QARGG. A caspase-9 homologue has not been characterised in any bivalve species to date. Only expressed sequence tags (ESTs) in *C. gigas* [12] and Manila clams, *Ruditapes philippinarum* [49] have been identified as caspase-9 homologues by automated annotation without detailed analysis of sequences or phylogenetic relationships, and ESTs annotated as caspase-9 homologues were not described for the oyster *Ostrea edulis* [13]. It appears that bivalves might be missing a direct homologue to vertebrate caspase-9, which seems surprising given that caspase-9 plays an important role in the intrinsic apoptotic pathway forming apoptosomes with Cyt-c and Apaf-1 proteins for further downstream activation of executioner caspases (Fig. 1a). Additional bivalve genomes need to be searched for potential caspase-9 homologues to shed light on this missing caspase type. Interestingly, caspase-9 activity was detected in *C. gigas* haemocytes after UV irradiation that initiated apoptosis in haemocytes by using a vertebrate caspase-9 activity assay [50]. Caspase-9 activity was increased in the presence of *C. gigas* Cyt-c and inhibited by vertebrate caspase-9 inhibitor Z-LEHD-FMK, suggesting that a caspase-9-like homologue is indeed expressed in the Pacific oyster. Caspase-9 activity was also required for a successful caspase-3 activation, suggesting a functional cascade of initiator and executioner caspases for apoptosis initiation in the oyster. Without a clear candidate as a caspase-9 homologue in the oyster genome, it is possible, that one of the identified *C. gigas* caspase-2 homologues might function in a caspase-9-like manner. *C. gigas* possesses seven caspase-2 or caspase-2-like homologues, which is more than is reported for any other species. Further research is needed to explore whether one of these *C. gigas* caspase-2 homologues operates similarly to vertebrate caspase-9, including the ability to hydrolyse LEHD sequences of substrates, which seems to be specific for vertebrate caspase-9 homologues [20]. Apoptosome formation in *C. gigas* also appears to differ from vertebrates apoptosomes (caspase-9, Cyt-c and Apaf-1 proteins). Firstly, no direct Apaf-1 homologue could be identified in a BLAST search of the Pacific oyster genome, nor has an Apaf-1 homologue been characterised in detail in any other bivalve species. Secondly, a recent study reported that the Apaf-1 protein in oysters, for which unfortunately no sequence ID was provided, is missing the CARD domain and only contain WD-domains for binding Cyt-c [50]. In vertebrates, the Apaf-1 CARD domain binds to the caspase-9 CARD domain during apoptosome formation. A comprehensive *in-silico* analysis of apoptosome forming proteins across metazoan species has shown that although apoptosomes appear early in metazoan evolution, species-specific or taxa-specific deletion and duplication events could lead to unique types of apoptosome formations [51]. Combining these finding, it is possible that the bivalve mitochondrial pathways, in contradiction to what has been previously reported, may differ from their vertebrate counterparts.

With several caspase-2 homologues present in bivalves, this expansion of caspases might take part as initiators of programmed cell death along the two apoptotic pathways. Vertebrate caspase-2 pathways are not fully resolved, but caspases-2 form PIDDosomes by binding to cytoplasmic p53-induced proteins with death domains (PIDD) and bipartite adapter RIP-associated Ich-1/Ced-3-homologue proteins with death domains (RAIDD) as part of the intrinsic apoptotic pathway (Fig. 1a) [9]. PIDD protein homologues have been reported in the genome of the clam species *Mya arenaria* [7] and *R. philippinarum* [49]. A RAIDD homologue has also been identified in the mussel *Mytilus coruscus* in the NCBI database (GenBank ID: CAC5373468.1), thus supporting functional bivalve caspase-2 homologues including formation of PIDDosomes. RAIDD adaptor binding to caspase-2 might also be involved in a more direct response with TNF-receptors for the extrinsic pathways interacting with executioner caspases. Caspase-2 regulated apoptosis in vertebrates has implications for host-pathogen interactions as well as in responses to endoplasmic reticulum stress and DNA damage [52]. Bivalve caspase-2 homologues show similar implications with upregulated expression of Mg2-like and Cg2B in haemocytes after UV treatment [26] or in bacterial challenges [12], respectively. Increase in gene expression of Mg2-like also suggests that even caspase members without the conserved sites in the p20 subunit might fulfil an essential function during immune response. Caspase-2 homologues might also be involved in inhibition of apoptosis. A *C. gigas* inhibitor of apoptosis protein CgIAP2 has been shown to inactivate Cg2A [53]. This might be of particular interest for host-pathogen interactions where pathogens increase the host’s IAP expression to prevent apoptosis as suggested for *O. edulis* [54]. The surge in *C. gigas* caspase-2 homologues also indicates that more caspase-2 homologues might exist for *Crassostrea angulata* and *M. galloprovincialis* than the two previously characterised.

One of the most conserved caspase groups is the initiator caspase-8 group containing DED motifs in the prodomain. This group is also highly conserved in bivalves. To transmit apoptotic signals in vertebrates, caspase-8 forms a complex with membrane-bound death receptors (i.e. Fas, TNF or TRAIL) binding to Fas-associated proteins with DD motifs (FADD). This complex recruits procaspases-8 via a DED motif between FADD and the caspase prodomain. Caspase-8 then undergoes self-cleaving for activation. Many of these death receptor homologues have been described in bivalve species [1, 12, 55–64] as well as FADD homologues in different oyster species [12, 13, 57]. Based on previous research, caspase-8 homologues seem to be functional and operate in a vertebrate-like manner. Ch8A [29] and Cg8B [25] were both located in the cytoplasm after cell death was induced. Furthermore, Ch8A [29] and Mc8A [27] were able to activate human caspase-3 in transfected cells, supporting a downstream activation of executioner caspases by oyster and mussel caspase-8 homologues. Implications of caspase-8 in bivalve immune response, including potentially different functions and responses to pathogens, were provided by an increase in expression of Ch8A and Ch3/7 in haemocytes of *Crassostrea hongkongensis* oysters after challenge with *Vibrio alginolyticus* [29, 34]. Interestingly, the same *Vibrio* sp. did not induce the expression of the *C. gigas* Cg8B in Pacific oyster haemocytes [25]. This could either be a species specific or a caspase-type specific response, given that Ch8A and Cg8B are grouped into two different paralogous groups.

Overall, we identified three types of caspase-8 homologues in bivalves: [1] caspases with two DED motifs [2]; with two DED motifs and an additional DD motif, and [3] with only one DED motif in their prodomains. The structural divergence between these caspases is likely to be caused by gene duplication events leading to loss or gain of functional domains given that each of these domains correlates with one exon. How these variations in prodomain motifs affect their binding ability to FADD or death receptors is not clear. In vertebrates, FADDs interact with only one of the caspase-8 DEDs [65], suggesting that one-DED-containing caspase-8-like homologues of bivalves are potentially able to bind to FADD. Furthermore, the DD motif of Mc8B is able to bind to the DD motifs of human Fas receptors and FADD proteins [27]. We also identified a DD motif in Cg8B caspase that was not previously reported [25], suggesting that the additional DD motif is potentially common in this bivalve caspase-8 B group. In mammals, the second DED motif in the caspase-8 prodomain has a role in inhibiting apoptosis by inactivating procaspases-8/FADD complex through binding to a DED motif containing inhibitor c-FLIP [66]. Although no FLIP homologues have been identified in bivalves, the second DED motifs in caspase-8A and 8B groups might still be utilised for apoptosis regulation. Interestingly, FLIPs (v-FLIPs) have been identified in herpes viruses as a viral inhibitor with v-FLIPs inhibiting apoptosis of host cells when infecting cells [67]. Thus, the presence of only one DED motif containing bivalve caspase-8 homologues might be an adaptation to viral infection that utilises v-FLIPs to prevent host cells of apoptotic immune responses.

The NF-ĸB pathway in vertebrates is involved in the transcription of pro-inflammatory interleukins and cytoplasmic Nod-like receptors (NLRPs), which take part in the pyroptotic inflammatory response together with caspase-1 and other inflammatory caspases [10]. In *C. hongkongensis,* Ch8A has been shown to activate the NF-ĸB transcription factor [29]. Furthermore, FADD-caspase-8 complexes are also involved in activating and regulating inflammation [11, 14], thus, it is likely that bivalve caspase-8 homologues are also potentially involved in inflammation responses. However, cleaving of interleukins and induction of pyroptosis by inflammasomes including caspase-1 might differ between bivalves and vertebrates given that our BLAST search of the *C. gigas* genome did not identify an inflammatory caspase homologue (caspase-1/− 4 or − 5) nor was one reported previously for any other bivalve species. The previously characterised *C. gigas* caspase-1 homologues [31] did not show any characteristics of vertebrates caspase-1. This *C. gigas* caspase homologue, renamed Cg3B, was able to cleave DEVD and DMQD tetrapeptide sequences, which are specific to caspase-3-like caspases, instead of the sequence YVAD, the tetrapeptide substrate sequence specific to vertebrate caspase-1. Moreover, a gasdermin D homologue could not be identified in the Pacific oyster genome, nor has a gasdermin D homologue been reported for any other bivalve species. In vertebrates, gasdermin D is cleaved by caspase-1 to catalyse pyroptosis (Fig. 1B) [11, 24]. However, vertebrate pyroptosis can also be induced via a caspase-3 and gasdermin E pathway [11]. Recently, gasdermin E-like homologues were described for corals and other invertebrates including a gastropod species [68]. This study has shown that coral gasdermin E could be cleaved by human caspasec-3 and -7 as well as a proposed coral caspase-3 homologue, which induced pyroptosis in transfected HeLa cells. Furthermore, caspase-3 and gasdermin E coexpression could be linked to *Vibrio-*induced pyroptosis in corals. The Pacific oyster genome also possesses two uncharacterised proteins, which display gasdermin E-like structures and close phylogenetic relationships to other invertebrate gasdermins E (Additional file 3), supporting a similar pyroptosis pathways for bivalves. Thus, while it seems that bivalve pyroptosis and inflammation response is less likely regulated via a vertebrate-like caspase-1 pathway, a conserved pyroptosis induction pathway regulated by caspase-3 and gasdermin E-like proteins is probable.

The expansion of the executioner caspases in bivalves provides numerous caspase homologues for potential apoptotic regulation and execution, including possible involvement of several executioner caspases with distinct functions. In vertebrates, executioner caspases such as caspase-3, − 6 and − 7 are also functionally distinct, interacting with each other, cleaving different substrates, or displaying different cleaving efficiencies for the same substrate [69]. However, bivalve execution of apoptosis seems to differ from the vertebrate model given that no direct homologues to vertebrate’s executioner caspases have been identified. Instead, bivalve appear to possess their own specific groups, which may in some cases even be species-specific. The bivalve caspase-3A group, which is the most closely related group to vertebrate caspase-3 and -7, varies in the p20 conserved active site. However significant changes in gene expression patterns of Cg3A during development (Fig. 4), and Tg3A after cadmium exposure [35] were observed, thus suggesting that these two caspases might still fulfil essential functions in both development and immunotoxicity responses. This is supported by an increase in gene expression of a proposed caspase-3 homologue (94% identity with Cg3A) in *O. edulis* after infection with *Bonamia* leading to an increase in apoptosis in haemocytes [70]. Interestingly, the same study also reported an increase in a proposed caspase-2 homologue, but sequence analysis showed no resemblance to one of our bivalve caspase-2 homologues, but an 77% identity with a *C. gigas* CARD containing protein CgCARDDCP-1 instead [71].

Nevertheless, most prior research was conducted on Cg3B, for which no caspase homologue in another bivalve species has been identified. This *C. gigas* caspase was cloned several times independently, with different names assigned [12, 31, 32]; it was renamed Cg3B in this study. Additional studies on Cg3B have been conducted in relation to spatial distribution (caspase-1 [33]) and in response to bacterial challenge and development (caspase-3 [37]). A study in *C. gigas* haemocytes on FMRFamides, specific neuropeptides, assessed the expression of Cg3B after stimulation with CgFMRFamides twice as caspase-1 and caspase-3 based on the primer pair sequences provided [38], with both analyses showing significant increases of Cg3B after FMRFamide injection. Cg3B is expressed in the cytoplasm [31, 33] as well as in the nucleus [31], which is similar to vertebrate caspase-3 translocating from the pro-form cytoplasm to an active form in the nucleus [72]. Cg3C, on the other hand, was only detected in the nucleus. Cg3B and Cg3C also displays high proteolytic activity to DXXD like substrates, which is similar to vertebrate caspase-3 homologues [31–33], as well as Cg3B induced apoptosis insomuch as it cleaves the poly ADP-ribose polymerase, a DNA repair protein [32]. Furthermore; Cg3B display lipopolysaccharides (LPS) binding activity, although some discrepancy in the LPS recognition was described [31, 32]. Two Cg3C-like homologues with DSRM motifs in their prodomain were identified, which is a motif that has not been previously reported for any caspase, but potentially function as viral pathogen detector in bivalves. DSRM motifs are usually used for posttranslational modifications of proteins, but they also take part as sensors and modulators of innate immunity by recognising and degrading intracellular viral dsRNA [73, 74].

Localisation of Ch3/7 in cytoplasm and nucleus in HEK293T cells as well as apoptosis activity of Ch3/7 in haemocytes was confirmed with Ch3/7 RNAi decreased apoptotic rates and Ch3/7 expression [34]. Ch3/7 are also able to activate the NF-ĸB pathway and p53 pathway. p53 is of particular interest when cells experience stressful environmental conditions such as DNA damage, UV light or tumorous growth with p53 activation inducing apoptosis by blocking specific cell cycle pathways [75]. p53 members were identified in several bivalve species in relation to apoptosis and neoplasia [7, 76–78]. p53 pathway activation was also found by Ch8 homologue with an intermediate activation of human caspase-3 [29], suggesting that both *C. hongkongensis* caspases regulate apoptosis via a p53 pathway. Caspase-3 like activity was also reported for Cg3/7 with DEVDase activity [36], for which research suggested that the long intersubunit linker sequence of Cg3/7 found in many caspase-3/7 members is essential for maximal DEVDase activity. Increases in expression of Mg3/7 [26] and Tg3/7 [35] after apoptosis induction with UV light or cadmium further supports that this large bivalve caspase group fulfils important functions during apoptotic processes. Nevertheless, how the newly identified Cg3/7 members and the Cg3/7-like group are involved in apoptosis still needs to be clarified. Cg3/7A-J caspases contain the conserved histidine/cysteine residues for a potential functionality, but Cg3/7-like homologues are less conserved, and sequences seem to be unique, which might suggest a potential new type of caspase in metazoans. Close gene clustering of oyster caspases, such as the Cg3/7-like A-F subgroup on the pseudo-chromosome LG7, suggests several gene duplications events in bivalves. Further research is needed to identify if these newly identified caspase groups are specific to the Pacific oyster, or are also present in other bivalves. The presence of a Mg3/7_1, _2 and _4 sup-group without a corresponding *C. gigas* homologue indicates that even between bivalve species, unique caspase groups might have evolved.

### Caspases in bivalve development and immune system function

The role of caspases and apoptosis in organogenesis and embryonic/larval development is well studied for many invertebrate species [79, 80], and expressions of various initiator and executioner caspases have also been reported throughout embryonic and larval stages in bivalves [12, 25, 30, 33, 37]. Caspase-regulated programmed cell death might also play a role in metamorphosis, which marks the transition from a larval to juvenile life stage. During metamorphosis, bivalve larvae undergo a massive re-structuring wherein they lose larval organs such as the velum and foot (species specific) that are required for pelagic dispersal, in favour of adult organs such as gills that are suited to later more sessile life stages. The loss of larval organs is almost certainly regulated by caspase-dependent apoptosis, as seen for many other animals that undergo metamorphosis [79, 81, 82]. Our analysis of *C. gigas* caspases prior, during and post metamorphosis (spat, also called juveniles 24 h post metamorphosis induction) showed expression of the proposed initiator and executioner caspases. However, although each caspase displayed a unique expression pattern, none of the six tested caspases showed an upregulated transcription during the first 6 hpe after the metamorphosis-inducing epinephrine treatment, with most of the larvae displaying a decrease in their expression (Fig. 4). Several possible explanations may elucidate these findings. First, apoptosis during development could be a very localised event in certain tissues, as compared to apoptosis during an immune response which is very rapid, intense and unspecific. Apoptosis during metamorphosis might be slower and thus might not be significantly reflected in transcription patterns. Furthermore, caspases are usually present in the cell as inactive zymogens until needed; thus, a slow accumulation could take place over time. Moreover, our sampling time point might have been too early to capture the full apoptotic process for larval organ absorption. A previous study on caspases during metamorphosis in *C. gigas* showed that Cg3B and Cg3C are highly expressed 6–24 h and 6–48 h post settlement, respectively [33]. Although it is not specified how these authors defined ‘post settlement’ (metamorphosis is a gradual process that occurs over a period of 12–36 h), these expression patterns suggest caspase activity occurs late in the metamorphosis process. Caspase homologues in *C. angulata,* Ca2A and Ca3C, also showed expression peaks 6 h post metamorphosis, although once again the authors did not define how they determined ‘post metamorphosis’ which is problematic as a specific timepoint in the transition process would be difficult to identify, especially in a 6 h window. In-situ hybridisation of these two oyster caspases in larvae prior to metamorphosis suggested the presence of caspases in the velum and larval foot [30]. Finally, it is also possible that none of our six caspases are involved in metamorphosis, as indicated by their decline in expression. Further investigations regarding caspase expression and regulation during bivalve metamorphosis are needed to gain more insight into how caspases are involved in the transition from larval to spat. However, as our gene expression profile of the four different executioner caspases, Cg3A, Cg3B, Cg3C and Cg3/7 clearly demonstrated, selection of the specific caspase-3 homologue to investigate is important, as these can vary through significant down- or upregulation before and after metamorphosis.

## Conclusion

Apoptosis in bivalves is a complex process, where most of the participating proteins, interactions and pathways remain unidentified. Our phylogenetic analysis of the 22 previously identified bivalve caspases, as well as the 35 previously unidentified *C. gigas* caspases based on our blasting of the Pacific oyster genome, shed light on the complexity of apoptotic and inflammatory pathways in this class of molluscs. In contrast to previous theories, bivalve apoptotic and inflammatory pathways appear to differ from the well-characterised vertebrate pathways given that no direct homologues to essential caspase such as caspase-9, caspase-1 or individual executioner caspase-3, − 6 and − 7 could be identified. Moreover, bivalves possess additional members of existing and novel caspase groups for both initiator and executioner caspases, including unique motifs in the caspase prodomains, further supporting a more unique function of caspases in regards to programmed cell death. This could be of particular interest in relation to the function of the bivalve immune system. Without the complex adaptive immune system that is present in vertebrates, most protostomian invertebrates have to rely on other regulatory mechanisms to regulate their innate immune responses to pathogens. The presence of several unique caspases could provide an additional regulatory instrument to respond to the large variety of pathogens, but also to environmental stressors that bivalve, especially more sessile species are increasingly exposed too. The presented phylogenetic analysis of bivalve caspases also provides a foundation for additional caspase-related research in bivalves or other lesser studied invertebrates. Automated annotation and poorly conducted phylogenetic analysis, including insufficient numbers of characterised homologues, can lead to inaccurate classification of newly identified proteins and incorrect assumptions about potential functions. As such, we have shown that caspases in bivalves are substantially more multifaceted than was previously understood.

## Methods

### Identification bivalve caspases and phylogenetic analysis

Putative *C. gigas* caspases were identified by using tBLASTn and BLASTp searches of the Pacific oyster genome at NCBI (genome annotation release102) with protein sequences of human caspases (caspase-1 to − 10) used as template. Identified protein sequences were checked for caspases-specific domains by Conserved Domain Database at NCBI [83] and ScanProsite [84]. Each identified protein sequence was used in a BLAST search against vertebrate protein database to exclude homology to possible non-caspase proteins. Gene location analysis for each *C. gigas* caspase were conducted and aligned to the genome data and assembled pseudo-chromosomes [40].

The large p20 and small p10 domains of the 35 putative *C. gigas* caspases identified by a BLAST search, together with the 23 previously identified caspases in bivalves, were aligned with caspases homologues (caspase-1 -caspase-10) of vertebrates and other invertebrate species using the default parameter of MUSCLE v3.8.31 [85] and edited manually in case of errors. Representatives of caspase homologues to vertebrate caspase-11 – caspase-16 or tunicates caspase-17 – caspase-22 were excluded as previous research has shown that these caspases are specific to these subphyla [86, 87]. For those putative caspases, which did not contain a small p10 domain, remaining protein sequence downstream to the large p20 domain were used in the alignment instead. A full list of all protein sequences used in the BLAST search and phylogenetic analysis are provided in Additional file 1. The alignment was trimmed with the intersubunit linker and insertions of single proteins were removed (Additional files 4 & 5). Two main trees were constructed for the proposed initiator and executioner caspases, pre-selected based on presence of CARD and/or DED containing prodomains, and a rough preliminary maximum likelihood tree containing all sequences. Two separate trees were constructed using the full sequence of CARD or DED domains (alignment Additional files 6 & 7). For each tree a Maximum Likelihood (ML) and a Bayesian Inference (BI) analysis were constructed. Models of protein sequence evolution for ML (AIC criteria) and BI (BIC criteria) analyses were estimated with ProtTest v2.4 [88] including proportion of invariable sites (+I), amino acid frequencies (+F) and gamma shape (+G): matrices LG + I + G + F (ML & BI initiator caspases, ML DED domains), LG + G (ML & BI executioner caspases, BI DED domains) and Blosum62 + G (ML & BI CARD domains). The ML analyses were constructed using PhyML v3.1 [89] and 1000 bootstrap replicates. The BI trees were calculated using MrBayes v3.2.7 [90] with four randomly started simultaneous Marcov chains running for two million generations, chains sampled every 100 generations and a burn-in of 5000 trees.

### Animal husbandry and metamorphosis assay

Pacific oyster, *C. gigas,* larvae were cultured at the South Australian Research and Development Institute in Adelaide, South Australia with larvae derived from 19 families, reared in 1 μm filtered seawater (FSW) at 24.5 ± 0.5 °C with a salinity of 36.5 ± 0.5 ppt as previous described [91]. Larvae were fed daily with an algal mixture of *Tisochrysis lutea*, *Pavlova lutheri*, *Chaetoceros muelleri*. and *Chaetoceros calcitrans.*

At 17 dpf and with a shell length of 300–330 μm, approx. 250 larvae were exposed to epinephrine hydrochloride (Sigma-Aldrich) at a concentration of 10^− 4^ M in a total volume of 2.5 ml FSW in glass shell vials (outside diameter x height: 29 × 94 mm). Exposure was terminated after 3 h by removing the EPI-containing seawater and adding 10 ml fresh FSW with algal feed. Larvae treated similarly, but without epinephrine exposure, were used as controls. After 24 h, metamorphosed individuals were assessed for the three replicates per treatment using an inverted microscope by counting live larvae, dead larvae and spat (juveniles with clear adult shell growth). No significant mortality was recorded for any biological replicate of the assay. Metamorphosis success was calculated as a percentage of larvae that successfully completed metamorphosis, with significant differences observed between EPI-treated and untreated individuals as assessed using a t-test in R v4.02 with significance at a probability level of 0.05.

### Gene expression analysis

The gene expression of 6 *C. gigas* caspases were evaluated in oyster larvae during late larval development (14 dpf, 15 dpf, 16 dpf), prior to metamorphosis induction (17 dpf), 3 hpe and 6 hpe to a 3 h epinephrine exposure, as well as in spat 24 hpe. For each sample point, three biological replicates were preserved in PaxGene Tissue system (PreAnalytix) and stored at − 20 °C.

Primers for Cg2A, Cg8B, Cg3/7, Cg3A, Cg3B, and Cg3C were designed with Primer Blast at NCBI [92] with an amplicon length ranging from 150 to 196 base pairs (bp). The elongation factor-1 α, ribosomal protein S18, ribosomal protein L7 were chosen as reference genes as previously described [91, 93]. Primer pairs were optimised for final concentrations, annealing temperatures and MgCl_2_ concentrations (Additional file 8); their specificities were verified by sequencing. Total RNA was extracted from all biological replicates per sample points (~ 20–40 mg per sample) using TRI Reagent RNA Isolation Reagent (Sigma-Aldrich) following the manufacturer’s protocol. Genomic DNA was removed using RQ1 RNase-Free DNase (Promega). One μg of clean total RNA was reverse-transcribed to cDNA using oligo (dT)18 primers with the High Capacity cDNA Reverse Transcription Kit (Applied Biosystems). The Luminaris Color HiGreen qPCR Master Mix (Thermo Scientific) in 10 ml reaction volume with 0.5 μl cDNA was used for the quantitative PCR (qPCR) reactions, which were run in duplicates on a 384 well plate PCR thermal cycler Light Cycler 480 Instrument II (Roche). The qPCR conditions were as follows: 95 °C for 10 min, 45 cycles of 95 °C for 15 s, 60–62 °C (primer pair dependent) for 30 s and 72 °C for 30 s. A melt curve was run at the end at 65–95 °C with a temperature transition rate of 0.05 °C. A non-template control and a cDNA dilution series for each primer pair were analysed in parallel to assess primer efficiency (standard curve) and exclude contamination. Primer efficiency and relative gene expression were based on a modified comparative Ct model as described in [94]. The average relative gene expression and standard error for each sample per caspase primer were calculated and significant differences between each sample point were assessed using a one-way ANOVA followed by multiple pairwise comparisons using a Tukey’s Honestly Significant Difference Test in R v4.02 with significance at a probability level of 0.05.

## Supplementary Information

**Additional file 1:.** Accession numbers and additional information about caspase protein sequences used for phylogenetic analysis of initiator and executioner caspase.

**Additional file 2: **Alignments of full protein and coding sequence CDS sequences of *Crassostrea gigas* caspase homologues: caspase-1 [12], caspase-1 [31], caspase-3 [32] and Cg3B (identified in this study).

**Additional file 3: **Phylogenetic relationship of the gasdermin family of *Crassostrea gigas* (CgGSDM1: XP_034300415; CgGSDM2: XP_034300423) and other invertebrates and vertebrates.

**Additional file 4:.** Alignment of CASc domain of initiator caspases.

**Additional file 5:.** Alignment of CASc domain of executioner caspases.

**Additional file 6:.** Alignment of CARD domains of initiator caspases.

**Additional file 7:.** Alignment of DED-DED domains of initiator caspases.

**Additional file 8: **Primers for quantitative gene expression analysis of caspases in *Crassostrea gigas*.

## Data Availability

The datasets used and/or analysed during the current study are mostly available in the Additional files. Additional datasets are available from the corresponding author on reasonable request.

## Abbreviations

Apaf-1: Apoptotic protease activating factor-1
BI: Bayesian Inference
CARD: Caspase-recruitment domain
CASc: Caspase-specific domain
Cyt c: Cytochrome c
DAMPs: Danger-associated molecular patterns
DD: Death domains
DED: Death-effector domain
dpf: Days post fertilisation
DSRM: Double stranded RNA-binding domains
EPI: Epinephrine
EST: Expressed sequence tags
FADD: Fas-associated protein with death domain
FSW: Filtered seawater
hpe: Hours post exposure start
IAP: Inhibitors of apoptosis
IL: Interleukins
LPS: Lipopolysaccharides
ML: Maximum Likelihood
MOMP: Mitochondrial outer membrane
NCBI: National Center for Biotechnology Information
NLRPs: Cytoplasmic Nod-like receptors
PAMPs: Pathogen-associated molecular patterns
PIDD: p53-induced protein with death domain
RAIDD: RIP-associated Ich-1/Ced-3-homologue protein with a death domain
ROS: Reactive oxygen species
