# Supplementary material for: Phylogenetic analysis of the caspase family in bivalves: implications for programmed cell death, immune response and development
Source: BMC Genomics. 2021 Jan 25;22:80. doi: 10.1186/s12864-021-07380-0 (PMC7836458; doi:10.1186/s12864-021-07380-0)
Supplement: Supplementary file 1 — Additional file 1:. Accession numbers and additional information about caspase protein sequences used for phylogenetic analysis of initiator and executioner caspase. [file 12864_2021_7380_MOESM1_ESM.pdf]

Additional File 1: Accession numbers and additional information about caspase protein sequences used for phylogenetic analysis of initiator and executioner caspase.

**Name**	**Species**	**GenBank ID**	**Previous names**<br><br>***Preliminary names based on automatic annotation***	**References**	**Potential isoform in *C. gigas* genome**
**<u>Initiator Caspases</u>**					
Aj2	*Apostichopus japonicus*	AOR82888			
Ca2	*Crassostrea angulata*	AGN75137	Ca2	[30]	
Cg2	*Crassostrea gigas*	XP_011419292	*CgDRONC*	*	
Cg2A	*Crassostrea gigas*	AEB54803<br>(AEB54804)<br>XP_011449817	Cg2<br><br>*Cg2-like*	[12]<br><br>*	
Cg2B	*Crassostrea gigas*	XP_011414267	*Cg3-like*	*	XP_034337253
Cg2C	*Crassostrea gigas*	XP_011414268	*Cg6*	*	XP_034337254
Cg2-like A	*Crassostrea gigas*	XP_034337082	*Cg1*	*	XP_034337083
Cg2-like B	*Crassostrea gigas*	XP_011423157	*Cg6*	*	XP_034337084
Cg2-like C	*Crassostrea gigas*	XP_011432762	*Cg8*	*	XP_011432763
DmDRONC	*Drosophila melanogaster*	Q9XYF4			
Dr2	*Danio rerio*	AAI46718	Dr2		
Hs2	*Homo sapiens*	P42575	Hs2		
Mg2-like	*Mytilus galloprovincialis*	ADZ24778	Mg2	[26]	
Xl2	*Xenopus laevis*	NP_001081404	Xl2		
CeCED3	*Caenorhabditis elegans*	P42573	CeCED3		
Hs9	*Homo sapiens*	P55211	Hs9		
Dr9	*Danio rerio*	NP_001007405	Dr9		
Xl9	*Xenopus laevis*	NP_001079035	Xl9		
Aj8	*Apostichopus japonicus*	AOR82886	Aj8		
Bf8	*Branchiostoma floridae*	ADB80148	Bf8		
Bl8	*Branchiostoma lanceolatum*	AEO22138	Bl8		
Cg8A	*Crassostrea gigas*	XP_034301972	*Cg8*	*	XP_034301971
Cg8B	*Crassostrea gigas*	AKP95634<br>XP_011445445	Cg8<br>*Cg8*	[25]<br>*	
Cg8-like A	*Crassostrea gigas*	XP_011414439	*Cg3-like*	*	XP_011414438<br>XP_011414436
Cg8-like B	*Crassostrea gigas*	XP_034334470	*Cg3-like*	*	XP_034334469

					XP_034334468
Cg8-like C	*Crassostrea gigas*	XP_034334496	*Cg3-like*	*	XP_034334497<br>XP_034334509
Ch8A	*Crassostrea hongkongensis*	AHB50667	Ch8	[29]	
DmDREDD	*Drosophila melanogaster*	Q8IRY7	DmDREDD		
Dr8	*Danio rerio*	AAS91706	Dr8		
Hd8	*Haliotis diversicolor*	AYK02334	Hd8		
Hd8-like	*Haliotis diversicolor*	ABY87390	abCaspase		
Hdd8	*Haliotis discus discus*	ADR78296	Hdd8		
Hl8	*Holothuria leucospilota*	ATE86972	Hl8		
Hs8	*Homo sapiens*	Q14790	Hs8		
Mc8A	*Mytilus californianus*	ADB80147	Mc8	[27]	
Mc8B	*Mytilus californianus*	ADB80146	Mc8-like	[27]	
Mco8A	*Mytilus coruscus*	AIS73194	Mco8	[28]	
Mg8A	*Mytilus galloprovincialis*	AIS73193	Mg8	[28]	
Mg8-like	*Mytilus galloprovincialis*	ADZ24779	Mg8	[26]	
Mm8	*Mus musculus*	CAA07677	Mm8		
Mt8	*Molgula tectiformis*	ABI64126	Mt8		
Tt8	*Tubifex tubifex*	ACP41139	Tt8		
Xl8	*Xenopus laevis*	NP_001079034	Xl8		
Dr10	*Danio rerio*	AWP39897	Dr10		
Hs10	*Homo sapiens*	Q92851	Hs10		
Xl10	*Xenopus laevis*	NP_001081410	Xl10		
**Inflammation caspases**					
Dl1	*Dicentrarchus labrax*	ABB05054	Dl1		
Xl1	*Xenopus laevis*	NP_001079341	Xl1		
Hs1	*Homo sapiens*	P29466	Hs1		
Mm1	*Mus musculus*	NP_033937	Mm1		
Hs4	*Homo sapiens*	P49662	Hs4		
Hs5	*Homo sapiens*	P51878	Hs5		
**Executioner caspase**					
Aj1	*Apostichopus japonicus*	QCB64985	Aj1		
Aj3	*Apostichopus japonicus*	AOR82887	Aj3		
Av3	*Anemonia viridis*	AAZ95018	Av3-like		
Bf3/7	*Branchiostoma floridae*	AAN45849	Bf3/7		

Bl3	*Branchiostoma lanceolatum*	AEO22140	Bl3-like		
Ca3C	*Crassostrea angulata*	AGN75138	Ca3	[30]	
Cg3/7	*Crassostrea gigas*	AMZ04158<br>XP_034299640	Cg37<br>*Uncharacterized protein*	[36]<br>*	
Cg3/7A	*Crassostrea gigas*	XP_011451826	*Uncharacterized protein*	*	XP_019929721
Cg3/7B	*Crassostrea gigas*	XP_011425040	*Cg3*	*	
Cg3/7C	*Crassostrea gigas*	XP_011454093	*Uncharacterized protein*	*	XP_019930379
Cg3/7D	*Crassostrea gigas*	XP_011454107	*Cg3-like*	*	XP_011454113<br>XP_034336797
Cg3/7E	*Crassostrea gigas*	XP_034313845	*Uncharacterized protein*	*	
Cg3/7F	*Crassostrea gigas*	XP_034309274	*Uncharacterized protein*	*	
Cg3/7G	*Crassostrea gigas*	XP_034309273	*Uncharacterized protein*	*	XP_019925280
Cg3/7H	*Crassostrea gigas*	XP_034331090	*Uncharacterized protein*	*	XP_034331089
Cg3/7I	*Crassostrea gigas*	XP_034331080	*Uncharacterized protein*	*	XP_034331081
Cg3/7J	*Crassostrea gigas*	XP_011443977	*Uncharacterized protein*	*	XP_034331079
Cg3/7K	*Crassostrea gigas*	XP_011423563	*Cg14*	*	
Cg3/7L	*Crassostrea gigas*	XP_034328252	*Cg7*	*	XP_034328251
Cg3/7-like	*Crassostrea gigas*	XP_034310204	*Cg7*	*	XP_034310205
Cg3/7-like A	*Crassostrea gigas*	XP_034302618	*Cg7*	*	XP_034302619
Cg3/7-like B	*Crassostrea gigas*	XP_034302611	*Cg7-like*	*	XP_034302612<br>XP_034302613
Cg3/7-like C	*Crassostrea gigas*	XP_034302616	*Cg7-like*	*	XP_011419037<br>XP_019920377
Cg3/7-like D	*Crassostrea gigas*	XP_034302615	*Cg7-like*	*	
Cg3/7-like E	*Crassostrea gigas*	XP_011419036	*Cg7*	*	XP_034302617
Cg3/7-like F	*Crassostrea gigas*	XP_034302622	*Cg7*	*	XP_034302623
Cg3/7-like G	*Crassostrea gigas*	XP_011426227	*Cg7*	*	XP_034306342<br>XP_034306343
Cg3/7-like H	*Crassostrea gigas*	XP_034306344	*Cg7*	*	XP_034306345<br>XP_034306346<br>XP_034306347<br>XP_034306348
Cg3A	*Crassostrea gigas*	XP_011445226	*Cg3*	*	XP_011445227<br>XP_019927950
Cg3B	*Crassostrea gigas*	AEB54802<br>(AEB54801)<br>EKC34324<br>AVH80607	Cg1<br>Cg3<br>Cg1<br>*Cg7-like*	[12]<br>[28]<br>[27]<br>*	 <br><br>XP_034310875

		XP_011447134			
Cg3B-like	*Crassostrea gigas*	XP_034311529	*Cg7*	*	
Cg3C	*Crassostrea gigas*	EKC43168<br>XP_011449627	Cg3<br>*Cg3*	[29]<br>*	
Cg3C-like A	*Crassostrea gigas*	XP_034304446	*Cg14*	*	
Cg3C-like B	*Crassostrea gigas*	XP_034304447	*Cg14*	*	
Ch3/7	*Crassostrea hongkongensis*	QHE23272	Ch3	[30]	
DmDAMM	*Drosophila melanogaster*	AAF82437	DmDAMM		
DmDCP1	*Drosophila melanogaster*	O02002	DmDCP1		
DmDECAY	*Drosophila melanogaster*	AAD54071	DmDECAY		
DmDRICE	*Drosophila melanogaster*	O01382	DmDRICE		
Ep3-like	*Exaiptasia pallida*	ABA62018	Ep-caspase-like		
Es37-like	*Eriocheir sinensis*	AGT29867	EsEffectorCaspase		
Es7-like	*Eriocheir sinensis*	AKS36883	Es7		
Hd3	*Haliotis diversicolor*	AYK02335	Hd3		
Lm1	*Locusta migratoria*	ATX63067	Lm1		
Mg3/7	*Mytilus galloprovincialis*	ADZ24782	Mg37_3	[26]	
Mg3/7_1	*Mytilus galloprovincialis*	ADZ24780	Mg37_1	[26]	
Mg3/7_2	*Mytilus galloprovincialis*	ADZ24781	Mg37_2	[26]	
Mg3/7_4	*Mytilus galloprovincialis*	ADZ24783	Mg37_4	[26]	
Mj1	*Marsupenaeus japonicas (=Penaeus japonicus)*	AZQ25116	Pj1		
Pm3-like	*Penaeus monodon*	ABI34434	PmCaspase		
Pme3-like	*Penaeus merguiensis*	AAX77407	PmeCaspase		
Sf1	*Spodoptera frugiperda*	P89116	Sf1		
Sm3	*Schistosoma mansoni*	ACU88129			
Sm7	*Schistosoma mansoni*	ACU88130	Sm7		
Tg3/7	*Tegillarca granosa*	AXN74785	Tg6	[35]	
Tg3A	*Tegillarca granosa*	AXN74784	Tg3	[35]	
Dr3	*Danio rerio*	NP_571952	Dr3		
Hs3	*Homo sapiens*		Hs3		
Mm3	*Mus musculus*	NP_033940	Mm3		
On3	*Oreochromis niloticus*	NP_001269823	On3		
Xl3	*Xenopus laevis*		Xl3		
Co7	*Cynops orientalis*	AFN55259	Co7		
Dr7	*Danio rerio*	NP_001018443	Dr7		
Hs7	*Homo sapiens*	P55210	Hs7		
Meg7	*Meleagris gallopavo*	XP_010713126	Meg7		

Mm7	*Mus musculus*	AAH05428	Mm7		
Ss7	*Salmo salar*	AAY28975	Ss7		
Xl7	*Xenopus laevis*	NP_001081408	Xl7		
Aj6	*Apostichopus japonicus*	AOR82885	Aj6		
Dr6	*Danio rerio*	NP_001018333	Dr6		
Hl6	*Holothuria leucospilota*	AUO29848	Hl6		
Hs6	*Homo sapiens*	P55212	Hs6		
Mm6	*Mus musculus*	O08738	Mm6		
On6	*Oreochromis niloticus*	XP_019215520	On6		

* NCBI *Crassostrea gigas* Annotation Release 102
