# Supplementary material for: Phylogenetic analysis of the caspase family in bivalves: implications for programmed cell death, immune response and development
Source: BMC Genomics. 2021 Jan 25;22:80. doi: 10.1186/s12864-021-07380-0 (PMC7836458; doi:10.1186/s12864-021-07380-0)
Supplement: Supplementary file 2 — Additional file 2: Alignments of full protein and coding sequence CDS sequences of Crassostrea gigas caspase homologues: caspase-1 [12], caspase-1 [31], caspase-3 [32] and Cg3B (identified in this study). [file 12864_2021_7380_MOESM2_ESM.pdf]

**Additional File 2:** Alignments of full protein (A) and coding sequence CDS (B) sequences of Crassostrea gigas caspase homologues: caspase-1 [12], caspase-1 [31], caspase-3 [32] and Cg3B (identified in this study).

## A: Protein sequence

```
Cg1_AEB54802_Zhang2011    1 MEEAM-SPECVSNTDVPDAKPVSETDAFPFGKSKSQS---GKPVATTIQTATEFFSHKYKMDYPNRGKAIIINNKKFNPNTGLNERNGTDQDASALCCRLSELDFDVDLF
Cg1_AEB54801_Zhang2011    1 MEEAM-SPECVSNTDVPDAKPVSETDAFPFGKSKSQS---GKPVATTIQTATEFFSHKYKMDYPNRGKAIIINNKKFNPNTGLNERNGTDQDASALCCRLSELDFDVDLF
Cg1_AVH80607_Lu2017       1 MEEAM-SPECVSNTDVPDAKPVSETDAFPFGKSKSQRKERGKPVATTIQTATEFFSHKYKMDYPNRGKAIIINNKKFNPNTGLNERNGTDQDASALCCRLSELDFDVDLF
Cg3_EKC34324_ Xu2016      1 MLLCLINGKQTADTDVPDAKPVSETDAFPFGKSKSQRKERGKPVATTIQTATEFFSHKYKMDYPNRGKAIIINNKKFNPNTGLNERNGTDQDASALCCRLSELDFDVDLF
Cg3B_XP_0114471           1 MEEAM-SPECVSNTDVPDAKPVSETDAFPFGKSKSQRKERGKPVATTIQTATEFFSHKYKMDYPNRGKAIIINNKKFNPNTGLNERNGTDQDASALCCRLSELDFDVDLF

Cg1_AEB54802_ Zhang2011 107 HNLKAEEIKVTLQKAAELDHRDNDCFTCAILSHGEDGFIWGIDRMIPINDLMEPFKGNKCLSLAGKPKIFFIQACRGTKFDDGVDMNVADAKGFMDVEPQFSLQKIPSEA
Cg1_AEB54801_ Zhang2011 107 HNLKAEEIKVTLQKAAELDHRDNDCFTCAILSHGEDGFIWGIDRMIPINDLMEPFKGNKCLSLAGKPKIFFIQACRGTKFDDGVDMNVADAKGFMDVEPQFSLQKIPSEA
Cg1_AVH80607_ Lu2017    110 HNLKAEEIKVTLQKAAELDHRDNDCFTCAILSHGEDGFIWGIDRMIPINDLMEPFKGNKCLSLAGKPKIFFIQACRGTKFDDGVDMNVADAKGFMDVEPQFSLQKIPSEA
Cg3_EKC34324_Xu2016     111 HNLKAEEIKVTLQKAAELDHRDNDCFTCAILSHGEDGFIWGIDRMIPINDLMEPFKGNKCLSLAGKPKIFFIQACRGTKFDDGVDMNVADAKGFMDVEPQFSLQKIPSEA
Cg3B_XP_0114471         110 HNLKAEEIKVTLQKAAELDHRDNDCFTCAILSHGEDGFIWGIDRMIPINDLMEPFKGNKCLSLAGKPKIFFIQACRGTKFDDGVDMNVADAKGFMDVEPQFSLQKIPSEA

Cg1_AEB54802_ Zhang2011 217 DFLIAYSVVPGYYSWRNSTNGSWFVQALSEVLMKHGQTVDLLTMMTRVNQIVANKFQSNTSHSDMNEKKQIPCVTSMLTKEVYFTSK
Cg1_AEB54801_ Zhang2011 217 DFLIAYSVVPGYYSWRNSTNGSWFVQALSEVLMKHGQTVDLLTMMTRVNQIVANKFQSNTSHSDMNEKKQIPCVTSMLTKEVYFTSK
Cg1_AVH80607_ Lu2017    220 DFLIAYSVVPGYYSWRNSTNGSWFVQALSEVLMKHGQTVDLLTMMTRVNQIVANKFQSNTSHSDMNEKKQIPCVTSMLTKEVYFTSK
Cg3_EKC34324_ Xu2016    221 DFLIAYSVVPGYYSWRNSTNGSWFVQALSEVLMKHGQTVDLLTMMTRVNQIVANKFQSNTSHSDMNEKKQIPCVTSMLTKEVYFTSK
Cg3B_XP_0114471         220 DFLIAYSVVPGYYSWRNSTNGSWFVQALSEVLMKHGQTVDLLTMMTRVNQIVANKFQSNTSHSDMNEKKQIPCVTSMLTKEVYFTSK
```

## B: CDS sequence

```
Cg1_HQ425704_Zhang2011    1 ATGGAAGAAG---CGATGAGCCCAGAGTGTGTTTCTAACACTGATGTTCCTGATGCTAAGCCTGTGTCAGAGACAGATGCCTTCCCATTCGGAAAATCAAAATCACAAAG
Cg1_HQ425703_Zhang2011    1 ATGGAAGAAG---CGATGAGCCCAGAGTGTGTTTCTAACACTGATGTTCCTGATGCTAAGCCTGTGTCAGAGACAGATGCCTTCCCATTCGGAAAATCAAAATCACAAAG
Cg1_KY807051_Lu2017       1 ATGGAAGAAG---CGATGAGCCCAGAGTGTGTTTCTAACACTGATGTTCCTGATGCTAAGCCTGTGTCAGAGACAGATGCCTTCCCATTCGGAAAATCAAAATCACAAAG
Cg3_CGI_1002342_Xu2016    1 ATGTTACTGTGTTTGATCAACGGCAAACAGACAGCTGACACTGATGTTCCTGATGCTAAGCCTGTGTCAGAGACAGATGCCTTCCCATTCGGAAAATCAAAATCACAAAG
Cg3B_XM_0114488           1 ATGGAAGAAG---CGATGAGCCCAGAGTGTGTTTCTAACACTGATGTTCCTGATGCTAAGCCTGTGTCAGAGACAGATGCCTTCCCATTCGGAAAATCAAAATCACAAAG

Cg1_HQ425704_Zhang2011  108 ---------TGGTAAACCAGTTGCCACTACAATACAGACAGCGACCGAGTTCTTCTCCCACAAGTACAAGATGGACTATCCAAACAGGGGTAAAGCTATCATCATCAACA
Cg1_HQ425703_Zhang2011  108 ---------TGGTAAACCAGTTGCCACTACAATACAGACAGCGACCGAGTTCTTCTCCCACAAGTACAAGATGGACTATCCAAACAGGGGTAAAGCTATCATCATCAACA
Cg1_KY807051_Lu2017     108 AAAAGAGCGTGGTAAACCAGTTGCCACTACAATACAGACAGCGACCGAGTTCTTCTCCCACAAGTACAAGATGGACTATCCAAACAGGGGTAAAGCTATCATCATCAACA
Cg3_CGI_1002342_Xu2016  111 AAAAGAGCGCGGTAAACCAGTTGCCACTACAATACAGACAGCGACCGAGTTCTTCTCCCACAAGTACAAGATGGACTATCCAAACAGGGGTAAAGCTATCATCATCAACA
Cg3B_XM_0114488         108 AAAAGAGCGCGGTAAACCAGTTGCCACTACAATACAGACAGCGACCGAGTTCTTCTCCCACAAGTACAAGATGGACTATCCAAACAGGGGTAAAGCTATCATCATCAACA

Cg1_HQ425704_Zhang2011  209 ACAAGAAATTTAACCCTAACACTGGGCTGAACGAGCGGAATGGCACGGACCAGGACGCCTCCGCTCTCTGCTGCCGTCTGTCCGAGCTCGACTTCGATGTAGATCTTTTT
Cg1_HQ425703_Zhang2011  209 ACAAGAAATTTAACCCTAACACTGGGCTGAACGAGCGGAATGGCACGGACCAGGACGCCTCCGCTCTCTGCTGCCGTCTGTCCGAGCTCGACTTCGATGTAGATCTTTTT
Cg1_KY807051_Lu2017     218 ACAAGAAATTTAACCCTAACACTGGGCTGAACGAGCGGAATGGCACGGACCAGGACGCCTCCGCTCTCTGCTGCCGTCTGTCCGAGCTCGACTTCGATGTAGATCTTTTT
Cg3_CGI_1002342_Xu2016  221 ACAAGAAGTTTAACCCTAACACTGGGCTGAACGAGCGGAATGGCACGGACCAGGACGCCTCCGCTCTCTGCTGCCGTCTGTCTGAGCTCGACTTCGATGTAGATCTTTTT
Cg3B_XM_0114488         218 ACAAGAAGTTTAACCCTAACACTGGGCTGAACGAGCGGAATGGCACGGACCAGGACGCCTCCGCTCTCTGCTGCCGTCTGTCCGAGCTCGACTTCGATGTAGATCTTTTT

Cg1_HQ425704_Zhang2011  319 CACAACCTGAAAGCAGAGGAAATCAAAGTTACACTACAGAAAGCCGCCGAATTGGACCACAGAGACAACGACTGTTTCACCTGCGCCATTCTGTCCCATGGGGAGGATGG
Cg1_HQ425703_Zhang2011  319 CACAACCTGAAAGCAGAGGAAATCAAAGTTACACTACAGAAAGCCGCCGAATTGGACCACAGAGACAACGACTGTTTCACCTGCGCCATTCTGTCCCATGGGGAGGATGG
Cg1_KY807051_Lu2017     328 CACAACCTGAAAGCAGAGGAAATCAAAGTTACACTACAGAAAGCCGCCGAATTGGACCACAGAGACAACGACTGTTTCACCTGCGCCATTCTGTCCCATGGGGAGGATGG
Cg3_CGI_1002342_Xu2016  331 CACAACCTGAAAGCAGAGGAAATCAAAGTTACACTACAGAAAGCCGCCGAATTGGACCACAGAGACAACGACTGTTTCACCTGCGCCATTCTGTCCCATGGGGAGGATGG
Cg3B_XM_0114488         328 CACAACCTGAAAGCAGAGGAAATCAAAGTTACACTACAGAAAGCCGCCGAATTGGACCACAGAGACAACGACTGTTTCACCTGCGCCATTCTGTCCCATGGGGAGGATGG

Cg1_HQ425704_Zhang2011  429 ATTTATATGGGGGATAGACAGAATGATCCCCATTAATGACCTGATGGAGCCCTTCAAAGGCAACAAATGTCTCTCTCTGGCAGGAAAACCCAAAATCTTCTTTATCCAGG
Cg1_HQ425703_Zhang2011  429 ATTTATATGGGGGATAGACAGAATGATCCCCATTAATGACCTGATGGAGCCCTTCAAAGGCAACAAATGTCTCTCTCTGGCAGGAAAACCCAAAATCTTCTTTATCCAGG
Cg1_KY807051_Lu2017     438 ATTTATATGGGGGATAGACAGAATGATCCCCATTAATGACCTGATGGAGCCCTTCAAAGGCAACAAATGTCTCTCTCTGGCAGGAAAACCCAAAATCTTCTTTATCCAGG
Cg3_CGI_1002342_Xu2016  441 ATTTATATGGGGGATAGACAGAATGATCCCCATTAATGACCTGATGGAGCCCTTCAAAGGCAACAAATGTCTCTCTCTGGCAGGAAAACCCAAAATCTTCTTTATCCAGG
Cg3B_XM_0114488         438 ATTTATATGGGGGATAGACAGAATGATCCCCATTAATGACCTGATGGAGCCCTTCAAAGGCAACAAATGTCTCTCTCTGGCAGGAAAACCCAAAATCTTCTTTATCCAGG

Cg1_HQ425704_Zhang2011  539 CTTGCCGTGGAACAAAGTTTGATGACGGCGTGGATATGAATGTCGCAGATGCTAAAGGCTTCATGGACGTAGAGCCTCAGTTTTCTCTCCAGAAGATCCCGTCAGAGGCC
Cg1_HQ425703_Zhang2011  539 CTTGCCGTGGAACAAAGTTTGATGACGGCGTGGATATGAATGTCGCAGATGCTAAAGGCTTCATGGACGTAGAGCCTCAGTTTTCTCTCCAGAAGATCCCGTCAGAGGCC
Cg1_KY807051_Lu2017     548 CTTGCCGTGGAACAAAGTTTGATGACGGCGTGGATATGAATGTCGCAGATGCTAAAGGCTTCATGGACGTAGAGCCTCAGTTTTCTCTCCAGAAGATCCCGTCAGAGGCC
Cg3_CGI_1002342_Xu2016  551 CTTGCCGTGGAACAAAGTTTGATGACGGCGTGGATATGAATGTTGCAGATGCTAAAGGCTTCATGGACGTAGAGCCTCAGTTTTCTCTCCAGAAGATCCCGTCAGAGGCT
Cg3B_XM_0114488         548 CTTGCCGTGGAACAAAGTTTGATGACGGCGTGGATATGAATGTCGCAGATGCTAAAGGCTTCATGGACGTAGAGCCTCAGTTTTCTCTCCAGAAGATCCCGTCAGAGGCC

Cg1_HQ425704_Zhang2011  649 GACTTCCTGATTGCTTACTCCGTTGTTCCAGGTTACTACTCCTGGAGGAACTCAACCAATGGCTCCTGGTTCGTCCAGGCGCTGTCTGAGGTCCTGATGAAGCATGGCCA
Cg1_HQ425703_Zhang2011  649 GACTTCCTGATTGCTTACTCCGTTGTTCCAGGTTACTACTCCTGGAGGAACTCAACCAATGGCTCCTGGTTCGTCCAGGCGCTGTCTGAGGTCCTGATGAAGCATGGCCA
Cg1_KY807051_Lu2017     658 GACTTCCTGATTGCTTACTCCGTTGTTCCAGGTTACTACTCCTGGAGGAACTCAACCAATGGCTCCTGGTTCGTCCAGGCGCTGTCTGAGGTCCTGATGAAGCATGGCCA
Cg3_CGI_1002342_Xu2016  661 GACTTTCTGATTGCTTACTCCGTTGTTCCAGGTTACTACTCCTGGAGGAACTCAACCAATGGCTCCTGGTTTGTCCAGGCGCTGTCTGAGGTCCTGATGAAGCATGGCCA
Cg3B_XM_0114488         658 GACTTCCTGATTGCTTACTCGGTTGTCCCAGGTTACTACTCCTGGAGAAACTCGACCAATGGCTCCTGGTTCGTCCAGGCGCTGTCTGAGGTCCTGATGAAGCATGGCCA

Cg1_HQ425704_Zhang2011  759 GACAGTGGACCTCCTGACCATGATGACACGTGTCAATCAAATCGTCGCCAACAAGTTCCAGTCTAACACCTCCCACTCCGACATGAACGAGAAGAAACAGATCCCATGTG
Cg1_HQ425703_Zhang2011  759 GACAGTGGACCTCCTGACCATGATGACACGTGTCAATCAAATCGTCGCCAACAAGTTCCAGTCTAACACCTCCCACTCCGACATGAACGAGAAGAAACAGATCCCATGTG
Cg1_KY807051_Lu2017     768 GACAGTGGACCTCCTGACCATGATGACACGTGTCAATCAAATCGTCGCCAACAAGTTCCAGTCTAACACCTCCCACTCCGACATGAACGAGAAGAAACAGATCCCATGTG
Cg3_CGI_1002342_Xu2016  771 GACAGTGGACCTCCTGACCATGATGACACGTGTCAATCAAATCGTCGCCAACAAGTTCCAGTCTAACACCTCCCACTCCGACATGAACGAGAAGAAACAGATCCCATGTG
Cg3B_XM_0114488         768 GACAGTGGACCTCCTGACCATGATGACACGTGTCAATCAAATCGTCGCCAACAAGTTCCAGTCTAACACCTCCCACTCTGACATGAATGAAAAGAAACAAATCCCCTGTG

Cg1_HQ425704_Zhang2011  869 TCACCTCCATGCTGACCAAGGAGGTTTACTTCACTAGCAAATAG
Cg1_HQ425703_Zhang2011  869 TCACCTCCATGCTGACCAAGGAGGTTTACTTCACTAGCAAATAG
Cg1_KY807051_Lu2017     878 TCACCTCCATGCTGACCAAGGAGGTTTACTTCACTAGCAAATAG
Cg3_CGI_1002342_Xu2016  881 TCACCTCCATGCTGACCAAGGAGGTTTACTTCACTAGCAAATAG
Cg3B_XM_0114488         878 TCACCTCCATGCTGACCAAGGAGGTTTACTTCACCAGCAAATAG
```
