# Supplementary material for: Phylogenetic analysis of the caspase family in bivalves: implications for programmed cell death, immune response and development
Source: BMC Genomics. 2021 Jan 25;22:80. doi: 10.1186/s12864-021-07380-0 (PMC7836458; doi:10.1186/s12864-021-07380-0)
Supplement: Supplementary file 3 — Additional file 3: Phylogenetic relationship of the gasdermin family of Crassostrea gigas (CgGSDM1: XP_034300415; CgGSDM2: XP_034300423) and other invertebrates and vertebrates. [file 12864_2021_7380_MOESM3_ESM.pdf]

Additional File 3: (A) Phylogenetic relationship of the gasdermin family of *Crassostrea gigas* (CgGSDM1: XP_034300415; CgGSDM2: XP_034300423; both on pseudo-chromosome LG7) and other invertebrates and vertebrates. Maximum Likelihood analysis with matric LG+G+F using PhyML with 1,000 bootstrap replicates (bootstrap support values as percentage). Protein IDs for gasdermin A-E and DFNB59 for *Homo sapiens* (HsGSDMA: AAI09198; HsGSDMB: AAH25682; HsGSDMC: NP_113603; HsGSDMD: NP_001159709; HsGSDME: NP_004394; HsDFNB59: AAI46939), *Xenopus tropicalis* (XtGSDME: XP_002933445; XtDFNB59: XP_017953002), *Cynoglossus semilaevis* (CsGSDME: XP_016893587; CsDFNB59: XP_008322774), *Branchiostoma floridae* (BfGSDME: XP_002587369v), *Orbicella faveolate* (OfGSDME: XP_020607257), *Pomacea canaliculate* (PcGSDME: XP_025094672); *Lingula anatina* (LaGSDME: XP_013387688), *Exaiptasia pallida* (EpGSDME: XP_020910462), *Hydra vulgaris* (HvGSDME: XP_012557585; HvDFNB59: CDG70293). Sequence information was based on information provided by [68] **B)** Alignment of full gasdermin and DFNB59 protein sequence using the default parameter of MUSCLE v3.8.31. Yellow bar representing the part of the *C. gigas* proteins which were classified as part of the Gasdermin family based on the Conserved Domain Database at NCBI.

A)

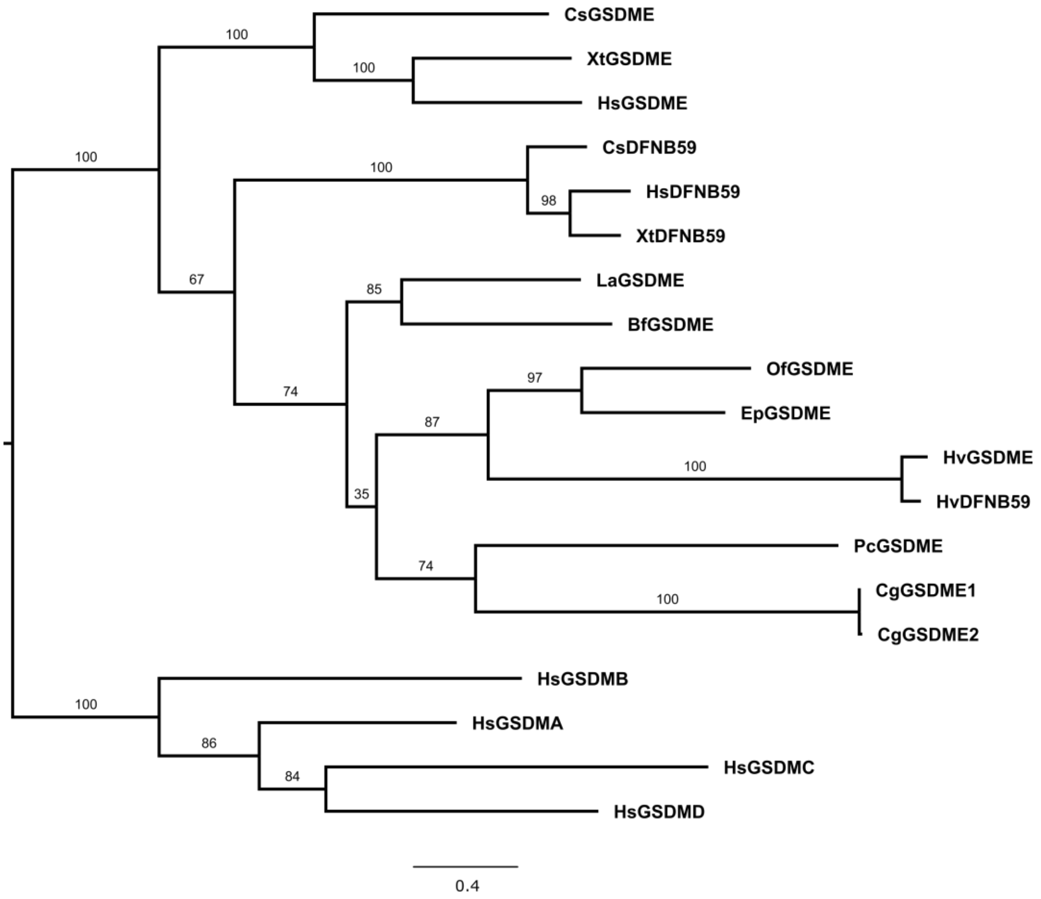

B)

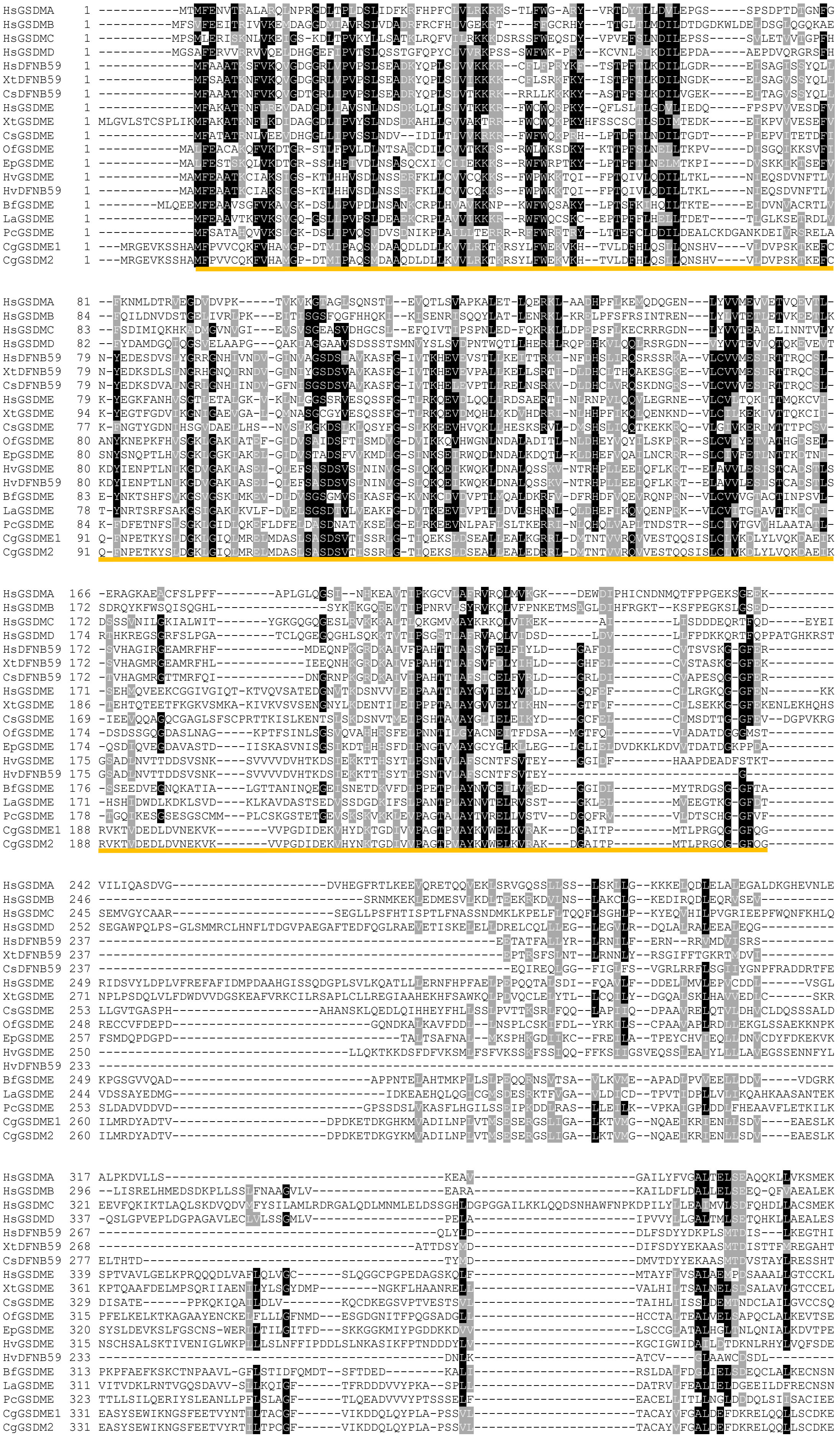

```
HsGSDMA  357 KILPV-QLKLVESTMEQN-FLLDKEGVFPLQPELLSSLGDEELTLTEALVGLSGLEVQRSGPQYMWDPDTLPRLCALYAGLSLLQQLTKAS-
HsGSDMB  353 GTLPL-LKDQVKSVMEQNWDELASSPPD-------MDYDPEARILCALYVVVSILLELAEGPTSVSS-------------------------
HsGSDMC  421 RILLQ-QQELVRSILEPN-FRYPWSIPFTLKPELLAPLQSEGLAITYGLLEECGLRMELDNPRSTWDVEAKMPLSALYGTLSLLQQLAEA--
HsGSDMD  396 QTLLG-PLELVGSLLEQS-APWQERSTMSLPPGL-LGNSWGEGAPAWVLLDECGLELGEDTPHVCWEPQAQGRMCALYASLALLSGLSQEPH
HsDFNB59 296 RVNLL-NHNIPKGPCIL--CGMGNFKRETVYGCF-QCSVDGQKYVRLHAV----------------------------PCFDIWHKRMK---
XtDFNB59 303 RVNLL-NNNIPKGPCAL--CGMGNSKRETVYGCF-ECSFNGQKYVRLHAV----------------------------PCFDLWHKRMK---
CsDFNB59 314 RVNLL-KHNISKGPCAL--CGMGNQKRETVYGCL-ECTSGGQKYVRLHVV----------------------------PCFDLWHKTIR---
HsGSDME  411 QIIPT-LCHLLRALSDDGVSDLEDPTLTPLKDT--ERFGIVQRLFASADISLERLKSSVKAVILKDSKVFPLLLCITLNGLCALGREHS---
XtGSDME  431 QLLPV-FSALMNMSSDEGLCSTTEPALMDFLDQ--ERFYVSQKLFALFNIELEIKEDFIYAATAEDPGFLPLILFIVITGLQLLKRD-----
CsGSDME  393 AELQT-MDQLVSCVSGKGELLLSSKDQTPLTE---DVFEKVVHLFASSNVSLRKDGDTVMTEINPEAGHAPLLLCIAVRALASLSVC-----
OfGSDME  390 YLEPL-LYLLKNVMYSKD-TSVDDPLLQRVWT---HSANPAKNLLLSLGFDQVITEGNNKLLQLTWDSEHISLEDTYVAVFVLCSE------
EpGSDME  394 YCDQL-LYLIRNAICGKN-TPADDPRLKRIYT---HSSNPGKSFLLALGF--QLTKQNGKDILALPPDLECPLEDAYVAVFVMAS-------
HvGSDME  393 HLKLL-LHIVESKILNKE---INEEIQINLMF---SENQSTKLFLNGIGF--VFETEEEKEVMTYPSEDHQKLLDAFINIYLLSA-------
HvDFNB59 251 ------LNKIDSTLTNKD---ILNEFPAAAWL------------------------------------------------------------
BfGSDME  380 FAPAI-LHVLEQGIAGNP-VPLDDPAISILYV---KSNNPGQDFIEAMDF---GIQDVGNKKALVTEDDGKTLYAAQQAVYGLWGGL-----
LaGSDME  380 LAKPL-LHVLNQALHGRD-VPLDD--IKALY----TDRNPAQSFLNEMDF-----HIKGLDTLNPPAGQSNKMEAAYIAVLALWG-------
PcGSDME  395 TGISQDILCLLRPVWDTDTREVEMPSDSCL-----LQTDTGKKLSQAFGF------EVTEFLLSLSEDNVRDVQALYLIIFLVVGSSDTQQQ
CgGSDME1 402 AGISL-IEICSQAISGET-AIVLPDFPKVLT-----KGTVGRDLAESLGF----QLNDDKNAITSPSQITKTLENSYWVLYALFK-------
CgGSDM2  402 AGISL-VEICSQAISGET-AIVLPDFPKVL-----TKGTVGRDLAESLGF----QLNDDKNAITSPSQITKTLENSYWVLYALFK-------
```
