# Supplementary material for: Phylogenetic analysis of the caspase family in bivalves: implications for programmed cell death, immune response and development
Source: BMC Genomics. 2021 Jan 25;22:80. doi: 10.1186/s12864-021-07380-0 (PMC7836458; doi:10.1186/s12864-021-07380-0)
Supplement: Supplementary file 4 — Additional file 4:. Alignment of CASc domain of initiator caspases. [file 12864_2021_7380_MOESM4_ESM.pdf]

Additional File 4: Alignment of CASc domain of initiator caspases. Light blue coloured: used alignment for phylogenetic analyses. Orange underscore: large p20 subunit, blue underscore: small p10 subunit, black underscore: intersubunit linker.

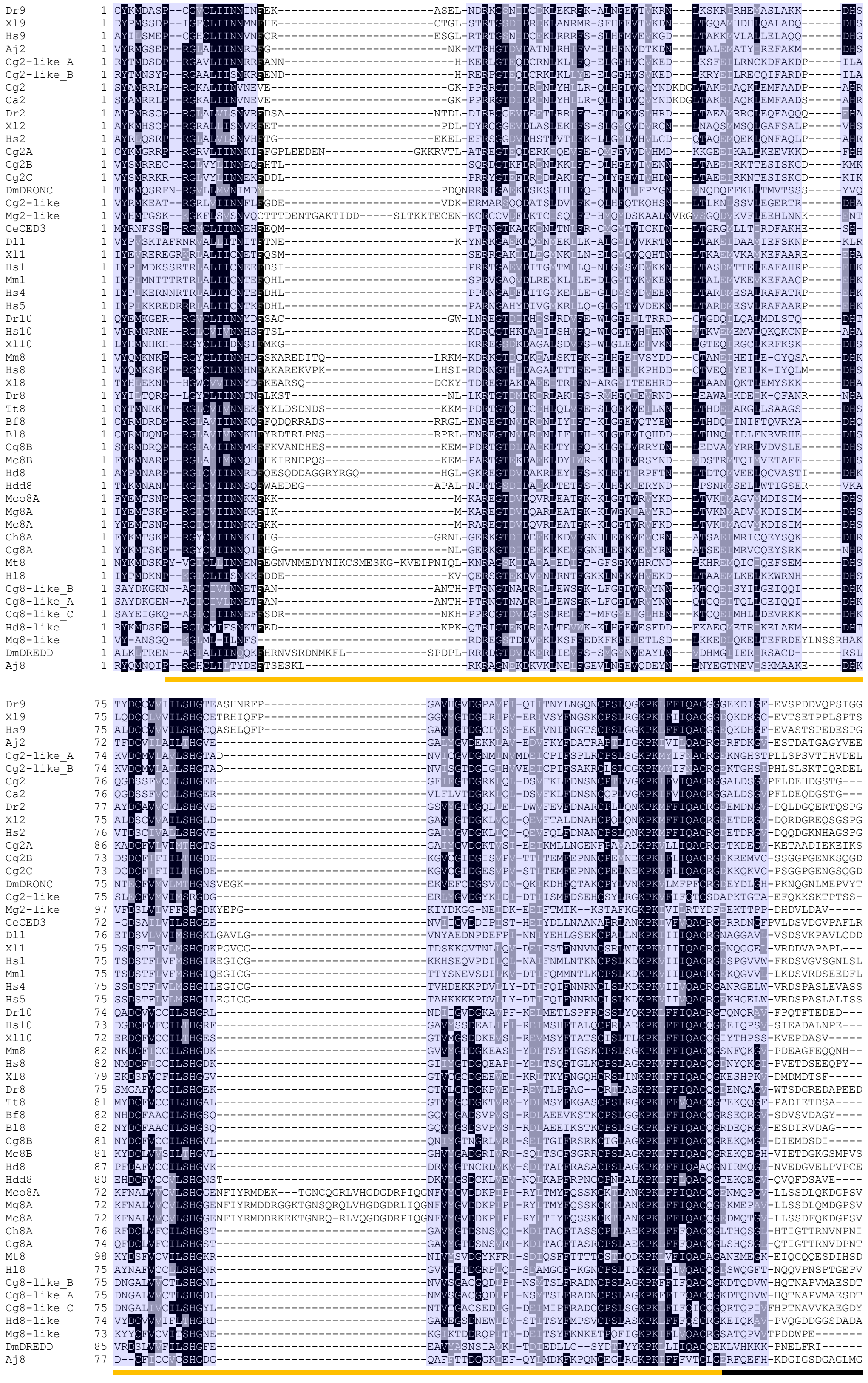

```
Dr9          159 IDDEMDAIPMSSSSD--------SLSTASDELDARASLP-TPSDILVSYS---TFPGYVSWRD--TEAGSWYVENLDRVLEENAI---TDDLVTMLMLVNDA--------
Xl9          159 TSLQSDATPVFSGE------------GDRDEVDAVSNIP-TPSDILVSYS---TFPGYVSWRD--KHTGSWYVEVLDSVLAEHAA---ADDLQSLLVMVADG--------
Hs9          159 SNPEPDATPFQEGL------------RTFDQLDAISSLP-TPSDIFVSYS---TFPGFVSWRD--PKSGSWYVETLDDIFEQWAH---SEDLQSLLLRVANA--------
Aj2          149 TAPPPRENGDQDANALVDSMLRLEFEAPDAFASARSKVP-SMSDMLIAYA---TVPGYVSWRN--SERGSWFIQALTEVILEYSQ---SEDLLSMLTKVNSK--------
Cg2-like_A   154 QRDGP-----------------------NEVGGYRSQVL-ENQDILFCYS---TFPGYASYRD--PDRGSWFIQEFVEVFHEQAC---DEHVMDMLTEVNKR--------
Cg2-like_B   154 QRDGP-----------------------YGDMDTISQVL-DNQDILICYS---TFPGYTSYRD--TERGNWFIQEFVDVFYEHAD---EEHVMDMLTEINKR--------
Cg2          151 ----------------------------------SKQLP-TQSDMIICFP---TQAGYYAWRN--RERGSWYIEALVQIFMKYAK---SEDICTMLHRVNLL--------
Ca2          151 ----------------------------------SKQLP-TQSDMIICFP---TQAGYYAWRN--RERGSWYIEALVQIFMKYAK---SEDICTMLHRVNLL--------
Dr2          154 CEQRDAGREGERDNRE--------KKEEKERERLRVKLP-QRSDMICGFA---TLKGTAAMRN--TKKGSWFIQELNTAIRQRAN---NTHLSDILVQVKHF--------
Xl2          152 CEQSDAGREDI-----------------------KVRLP-TQSDMICAYA---CLKGTVSLRN--TKRGSWFVQDLVSVFSQHSK---DTHVADMLVKVNAL--------
Hs2          153 CEESDAGKEKL----------------------PKMRLP-TRSDMICGYA---CLKGTAAMRN--TKRGSWYIEALAQVFSERAC---DMHVADMLVKVNAL--------
Cg2A         163 TGEILKDLSLNTV----------------SDSKSESVTA-TMSHMVVAMA---STLGMVSYRN--KIYGSWFLTAVAYVFAKYSH---TNDIHQMLTKVNQL--------
Cg2B         150 GGGDRVDGMNAPDINVER----------EPNIYATQTVH-SKSDILVAYS---TPEGSLSWRK--TDAGSWFMQAIVWIFKFESH---VKDLVEMLGKVNNV--------
Cg2C         150 GSGDHVDGMNASDGGW------------ELGISATQTVH-SKSDFLVAYS---TPEGSLSWRK--MDAGSWFMQAIVWVFKSESH---VKDLVEMLGKVNDI--------
DmDRONC      156 AQEEKWPDTQT-----------------EGIPSPSTNVP-SLADTLVCYA---NTPGYVTHRD--LDTGSWYIQKFCQVMADHAH---DTDLEDILKKTSEA--------
Cg2-like     151 ------------------------SSLTLEDSGD------LRADMFVVKA---TKDVEVSY-N--GVFGSRFIQSLVYLLRNLVC---EEDFEEIVKKINHL--------
Mg2-like     170 ----------------------------KSSVHFIKDKP-NVDDLFVVSSQPKTKKGPWVVGD--GMNGSYFIQALIHVFKNMAH---EKSFMEMMEEVNTC--------
CeCED3       149 RGWDNRDGPLFNFL----------GCVRPQVQQVWRKKP-SQADILIAYA---TTAQYVSWRN--SARGSWFIQAVCEVFSTHAK---DMDVVELLTEVNKK--------
Dl1          158 VNQLELSAGEENI-----------------EDDTLHFVH-KEKDFISLLS---STPDTVSYRQ--PDRGCFLIQYIVDVINTSAH---VDDIEELFRTVMQR--------
Xl1          154 ------------------------------EDDAVHHVQ-TETDSICFYS---STPDTASWRN--PTKGSVFIIRLIEKMNELAH---CQPLGDIFLEVQSS--------
Hs1          157 PTTEEF------------------------EDDAIKKAH-IEKDFIAFCS---STPDNVSWRH--PTMGSVFIGRLIEHMQEYAC---SCDVEEIFRKVRFS--------
Mm1          157 TDAIF-------------------------EDDGIKKAH-IEKDFIAFCS---STPDNVSWRH--PVRGSLFIESLIKHMKEYAW---SCDLEDIFRKVRFS--------
Hs4          157 QSSENL------------------------EEDAVYKTH-VEKDFIAFCS---STPHNVSWRD--STMGSIFITQLITCFQKYSW---CCHLEEVFRKVQQS--------
Hs5          157 QSSENL------------------------EADSVCKIH-EEKDFIAFCS---STPHNVSWRD--RTRGSIFITELITCFQKYSC---CCHLMEIFRKVQKS--------
Dr10         148 -------------------------VLASDAGVPRDSIP-EMADYLMAMS---TVPWYASYRD--KSKGTWFIQSLCDNLRLLV--PRGNDLLSILTKVNAD--------
Hs10         147 ----------------------------QAPTSLQDSIP-AEADFLLGLA---TVPGYVSFRH--VEEGSWYIQSLCNHLKKLV--PRMLKFLEKTMEIRGRKRTVWGAK
Xl10         144 ---------------------------PVEHKKYIVNVP-KEADFLLGMS---TVDGYAAYRH--KKYGSWYIQALCKNLVEMV--PRGEDILSILTKVNKD--------
Mm8          157 -------------------------TLEVDSSSHKNYIP-DEADFLLGMA---TVKNCVSYRD--PVNGTWYIQSLCQSLRERC--PQGDDILSILTGVNYD--------
Hs8          157 -------------------------LEMDLSSPQTRYIP-DEADFLLGMA---TVNNCVSYRN--PAEGTWYIQSLCQSLRERC--PRGDDILTILTEVNYE--------
Xl8          151 ----------------------------YEPDANGSHLP-LEADFLTAFA---TVEDYTSLRH--RENGSIYIQQLCKALTTYT----NQDLIDILTSVNSD--------
Dr8          150 -----------------------EKYEEDAGIIVLRKIP-IEADFLIGMA---TVEHYLSYRH--TKEGSIFIQELCKKMEELC--PKKEDMLSILTKVNFE--------
Tt8          154 -----------------------------CAPSEVETIP-NEADFLLGLA---TVPGYVSYRS--KTQGSWYITTLVSMLSRYAN---RYDLLSILTRVNER--------
Bf8          155 --------------------------GSDGLAAFQETIP-NEADFLLSYS---TIPGYVSYRS--KTYGSWFVTKLVEILDNMGD---RYDLLSILIKVNEE--------
Bl8          155 --------------------------HAQGLEAFQETIP-NEADFLLSYS---TIPGYVSYRS--KTQGSWFVTKLVETLDNMGD---RHDLLSILIKVNEE--------
Cg8B         153 ---------------------------EADNGRNSEMIP-DEADFVLGCA---TVPGYVSYRS--RSQGSWFVNKLVEMLDKFAY---RYDLLSILVKVNEE--------
Mc8B         158 DSS--------------------EERQDLNVDRLRELIP-DEADFLLGYA---TVPGYVSYRS--RSQGSWYINKLSQNLDKYAY---QHDLLSILIKVNEE--------
Hd8          164 RTPVDASVVRDDPPQEKPDK---QKWRSKGSPDERNIIP-DESDFLLGYA---TSPGYVSYRS--KRHGSWYIIRLAEVLEKYGD---RLDLLSCLMMVNDE--------
Hdd8         154 ------------------------------DDGPTVMVA-NEADFLVGYA---TAPGYVSYRS--KSKGSWYISKLTKMLDTHAH---TEDLLSILTKVNNE--------
Mco8A        177 DESINLDVAPLSENAE-------ETDAAPHASDNQKVIP-DESDFLLGYA---TVFGYVSYRS--RSQGSFYVKSLTENLDKYAD---TLDLQALMVKVNHD--------
Mg8A         180 DDSIDLDVAPVSENTE-------EKDAAPHASDNQKVIP-DESDFLLGYA---TVFGYVSYRS--RSQGSFYVKSLTENLDNHAD---TLDLQALMVKVTHD--------
Mc8A         179 DESIDLDVAPVSENAE-------ETDAAPHASDNQKVIP-DESDFLLGYA---TVFGYVSYRS--RSQGSFYVKSLTENLDKHAD---TLDLQALMVKVNHD--------
Ch8A         153 QTDVMQEPPRSEVNP-----------SDNQTSSAHKMIP-DESDFLLGYA---TVPGYVSYRS--RSQGSFYINILAEKLKKYALGPRGHDLVSILVSVNNA--------
Cg8A         151 QMDATQEPPRSEVNP-----------NEDRTSSAHKMIP-DESDFLLGYA---TVPGYVSYRS--RSQGSFYINILAEKLKKYALGPRGHDLVSILVSVNAA--------
Mt8          175 GD-----------------------------DNIKEATP-FLADFLLANS---TWPGYRSYRS--TKTGSWFITALVKNLKMYSC---KEDLVSIMTRTNND--------
Hl8          151 GM----------------------------GSIHTPQLP-NEADFVLLLA---TVPGFKSARS--SKKGSWFIMALTKILQTHVD---IE-IHSAMTLLNYE--------
Cg8-like_B   152 NLFPNSPMAEVMT--------------EVPHLHQLNLAP-AEADFLIAHS---TMPGYVSFRN---DKGSFFVQSLVKHLKAHS---PREDVISILVEVNRD--------
Cg8-like_A   152 NLVPN--MAEVMT--------------EVPHLHQLNLAP-AEADFLIAHS---TMPGYVSFRN---GKGSFFVQSLVKHLKAHS---PREDVISILVEVNRD--------
Cg8-like_C   153 NMTQDK--AEV----------------TDSPTLPRGPFL-AEADFLIAFA---TMRGYASYRN---ENGSYFIQSLVDLLRKRS---PSEDVVSILVEVNHD--------
Hd8-like     151 ---------------------------TETIVQANKMVA-PEADFFGFA---TVAGMQALRN--PETGSWFIQSLVQALQAST----KSDLDSIMTTVRGK--------
Mg8-like     144 ---------------------------------DTMSVP-HDADILIAYA---TTPGYKCFRTKYSDIGAWFIHVLLEEIEESYE---SDHLEDMLISVRHK--------
DmDREDD      154 --------------------------------DVTTVSPDQHIDMLRAMS---TVNGYAALRH--TQTGSWFIGSLCDAIDRRSA---SEHIADILTIVTNE--------
Aj8          152 --------------------------------KLSGLWA-KEADFCICLS---TVPGYANARD--PSKGTIYVQNVYECFKANYK---STGLIDMLNDVRER--------

Dr9          244 -VSQIS----------AKGLYKQMPGSFNFLR-KLLYFQSSQS----------------------------
Xl9          240 -VS-------------SKGTYKQIPGYFNFLR-KRFYFKTD------------------------------
Hs9          240 -VS-------------VKGIYKQMPGCFNFLR-KKLFFK----TS--------------------------
Aj2          242 -VAKAFESSS--------GKNKQMPAPVTMLT-KKLFFFPGYVRK--------------------------
Cg2-like_A   224 -VSQKNHTDYSS----EA-SCVQIPCPATSLT-KKWYLNPPFLGASLGH----------------------
Cg2-like_B   224 -VSKRNYKDNSS----E-DSCVQIPCPATSLT-KKWYMNPPPFATSFRRTIYY------------------
Cg2          210 -VSRKVSRCPQI----EMDSMSQMSEYKSSLRMPHLFFYPGIGCV--------------------------
Ca2          210 -VSRKVSRCPQI----EMDSMSQMSEYKSSLRMPHLFFYPGIGCV--------------------------
Dr2          239 -GYFH------------------------------------------------------------------
Xl2          222 -IKEREGHAPGT----EFHRCKEMSEYCSTLC-RDLYLFPGSPSNGLPK----------------------
Hs2          224 -IKDREGYAPGT----EFHRCKEMSEYCSTLC-RHLYLFPGHPPT--------------------------
Cg2A         240 -VSRGRAKDNK-----QNKEFVAISESSYTLS-KDLYFCPGYFIEEVPQSRIQQASF--------------
Cg2B         233 -VSKGKARD-------VGSHFVTVSKYESSLR-KKLFFFPGIYGDKRVHPKCI------------------
Cg2C         231 -VSKGKAKS-------GWGHYVTVSKYESSLR-KKLFFFPGIYGDKKVHTDCI------------------
DmDRONC      232 -VGNKRTKKG---------SMQTGAYDNLGFN-KKLYFNPGFFNE--------------------------
Cg2-like     214 -NVV---------------SEPEIVEYTSSFT-KKLFFNP-------------------------------
Mg2-like     238 -IMNAMVPKQGK------TSVAEVMILEHSVQ-KSLFFFPGLLDVPAVMMKVPGWARFWQLVENL------
CeCED3       232 -VACGFQTSQ------GSNILKQMPEMTSRLL-KKFYFWPEARNSAV------------------------
Dl1          234 -FEDF-----------PVPNKRQMPTKDRCTLTKRFYFFPGL-----------------------------
Xl1          217 -FKDK-----------SPNQYSQMPTQERSTMTKKFYLFPGY-----------------------------
Hs1          226 -FE-------------QPDGRAQMPTTERVTLTRCFYLFPGH-----------------------------
Mm1          225 -FE-------------QPEFRLQMPTADRVTLTKRFYLFPGH-----------------------------
Hs4          226 -FE-------------TPRAKAQMPTIERLSMTRYFYLFPGN-----------------------------
Hs5          226 -FE-------------VPQAKAQMPTIERATLTRDFYLFPGN-----------------------------
Dr10         217 -VSKKSD---------KSGLKKQMPQPEFSLT-RTVVFPPPGLP---------------------------
Hs10         221 QISATSL---------PTAISAQTPRPPMRRW-SSVS----------------------------------
Xl10         211 -VSLKED---------SEQKLKQMPQPAYTLL-KKLIFPVPDVPFRSTTCQENSP----------------
Mm8          226 -VSNKDD---------RRNKGKQMPQPTFTLR-KKLFFPP-------------------------------
Hs8          226 -VSNKDD---------KKNMGKQMPQPTFTLR-KKLVFP----S---------------------------
Xl8          215 -VANMLFRL-------WRKNVTQMPSFKSELR-KKLILPPPNAM---------------------------
Dr8          221 -VSKRIL-----------KGYKQMPEPRYTLT-KKLVLPID------------------------------
Tt8          218 -VSEAFTK--------EG-NLKQVPAPMFTLR-KKLYFC--------------------------------
Bf8          222 -VGKAIAKK-------GGGQFKQSPAPVATLR-KKIFFSFLREARKPQVIPGVRTVNTAV-----------
Bl8          222 -VGKAIARK-------GGGEFKQSPAPVATLR-KKIFFSFLREARKPQVVPRASAVNTAV-----------
Cg8B         219 -VGRANARL-------EGGVFKQIPAPLVTLR-KKLFFK--------------------------------
Mc8B         231 -VGRATANI-------DNGAYKQIPAPMFTLR-KRIIFR--------------------------------
Hd8          254 -VAKGNAMT-------KDGVFKQVPAPMFTLR-KKVNLTSD-----PMWFRSLSTINE-------------
Hdd8         217 -VGTEL----------GPYGQKQSPAPQFTLT-KRLIFKKLDSTA--------------------------
Mco8A        263 -VSENDIGGK------GEQAKKQVPMTQITLR-KQLFFSPDNVK---------------------------
Mg8A         266 -VSEENI---------GKQAKKQVPMTQITLR-RLLLFSPHNLK---------------------------
Mc8A         265 -VSENNIELVK-----GEQAKKQVPMTQITLR-KQLFFSPDNLK---------------------------
Ch8A         238 -VSELHYSNE------DGHVFKQVPAPQFTLR-RKVQFDKC------------------------------
Cg8A         236 -VSELHYSNE------DGHMFKQVPAPQFTLR-RRVQFDKC------------------------------
Mt8          239 -VS--------------KNMYVQMPMHTNTLR-KKVFFKMTHEYKEKIDRELASINPLTTVAVAEGFAASP
Hl8          215 -LSKVRDAKMD------GKEVSQTAFPCNTLR-KMLFLQQPVDCPPRV-----------------------
Cg8-like_B   230 -VSQTIGRTS------GGQIAAQIPEPKVRLT-KKFYLFPVETEV--------------------------
Cg8-like_A   228 -VSQTIGRTS------GGQIAAQIPEPKVRLT-KKFYLFPVETEV--------------------------
Cg8-like_C   227 -VSQKIGKTE------EGRYVAQIPESIIRLT-KKFFLHPMNSKA--------------------------
Hd8-like     216 -VNNRECETVD------GKFLRQTAEVRTTLL-KKLYF---------------------------------
Mg8-like     206 -LAIDEEWR--------QGDFCQMPCTWTTLT-KRLFFR--------------------------------
DmDREDD      216 -VSKKR----------GSNDESMVPNVKSTFR-QHVYFPPRL-----------------------------
Aj8          213 -TNEKLKQNPVQLKSYDSPICHISPT-ISTLL-RKVYFSS-------------------------------
```
