# Supplementary material for: Phylogenetic analysis of the caspase family in bivalves: implications for programmed cell death, immune response and development
Source: BMC Genomics. 2021 Jan 25;22:80. doi: 10.1186/s12864-021-07380-0 (PMC7836458; doi:10.1186/s12864-021-07380-0)
Supplement: Supplementary file 5 — Additional file 5:. Alignment of CASc domain of executioner caspases. [file 12864_2021_7380_MOESM5_ESM.pdf]

Additional File 5: Alignment of CASc domain of executioner caspases. Light blue coloured: used alignment for phylogenetic analyses. Orange underscore: large p20 subunit, blue underscore: small p10 subunit, black underscore: intersubunit linker.

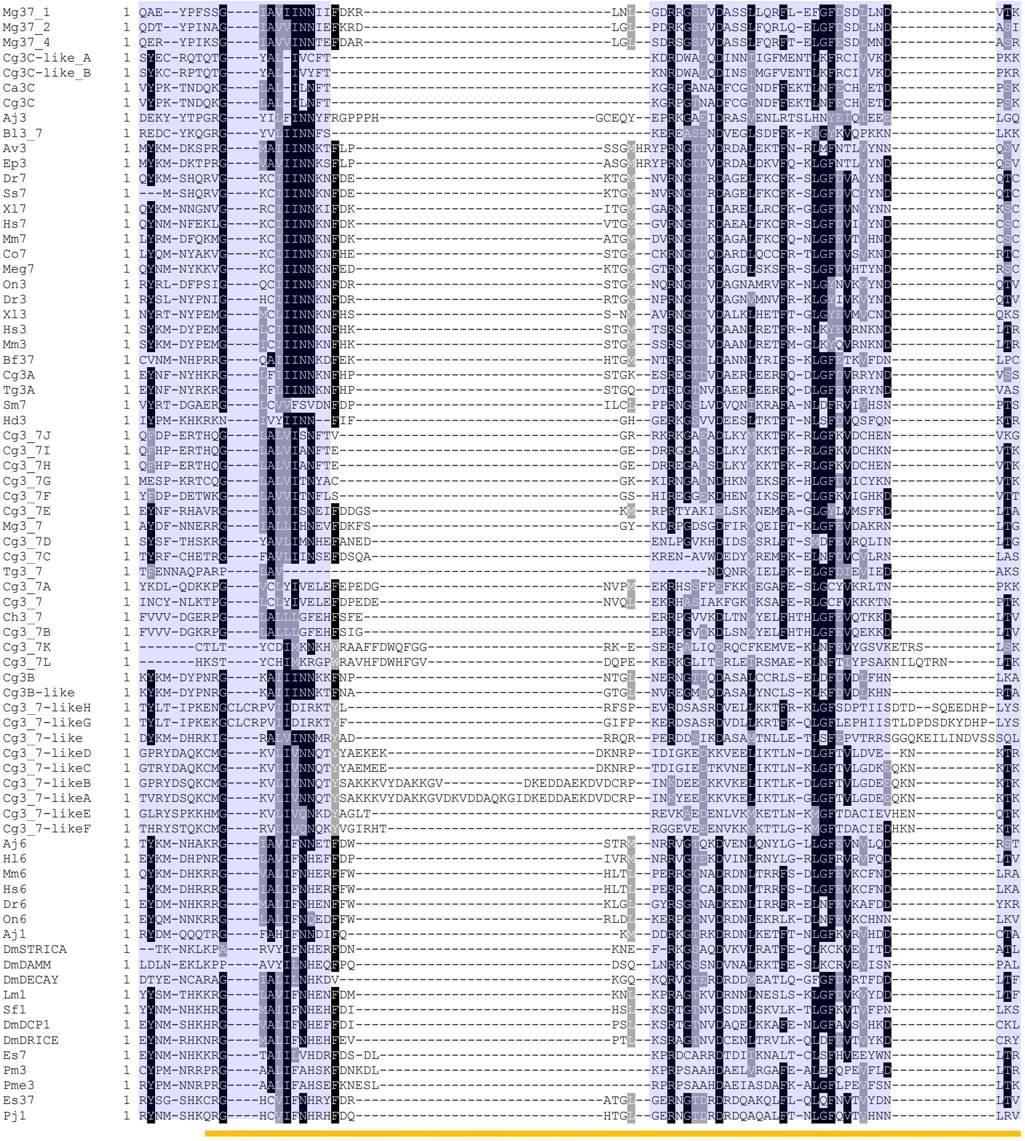

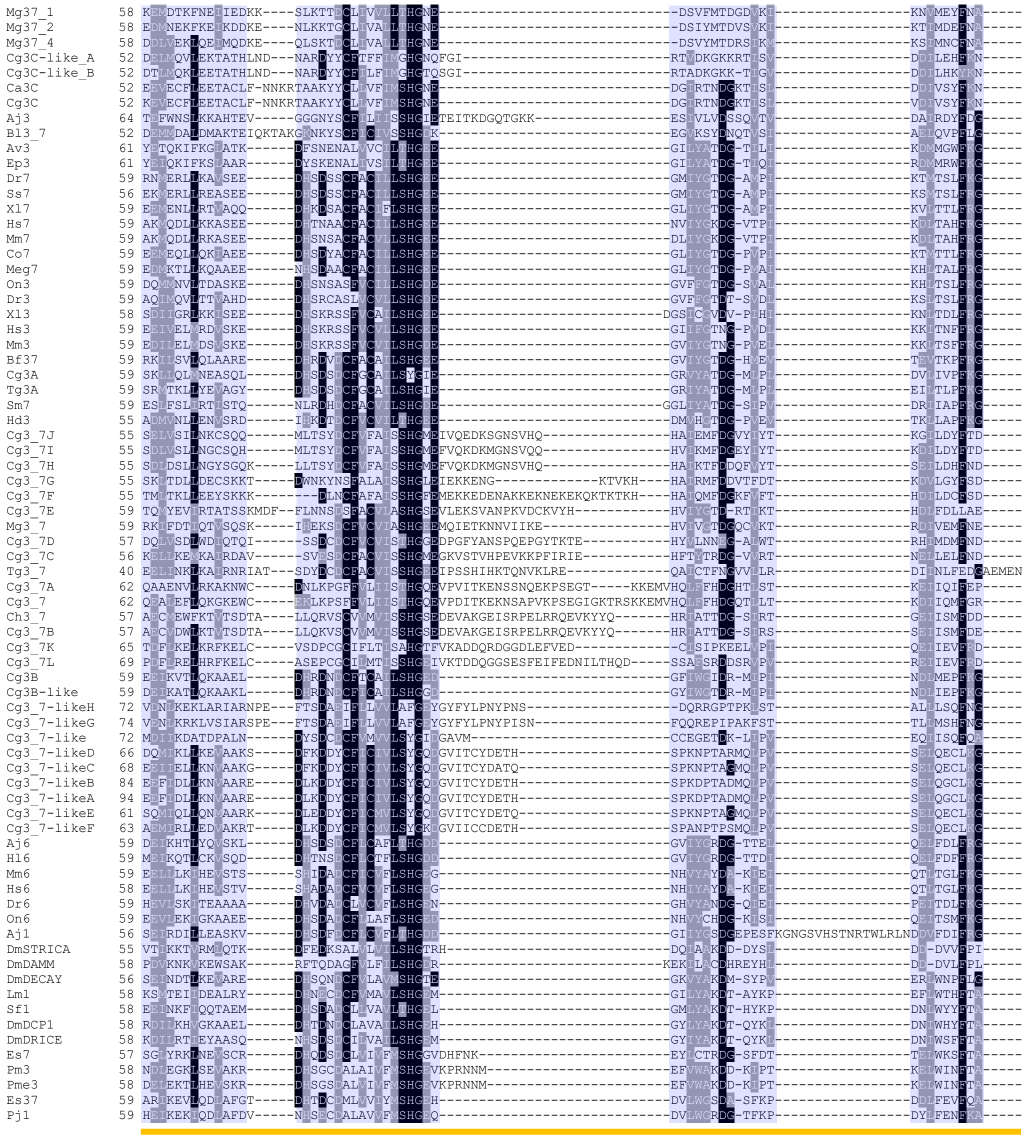
Mg37_1
Mg37_2
Mg37_4
Cg3C-like_A
Cg3C-like_B
Ca3C
Cg3C
Aj3
Bl3_7
Av3
Ep3
Dr7
Ss7
Xl7
Hs7
Mm7
Co7
Meg7
On3
Dr3
Xl3
Hs3
Mm3
Bf37
Cg3A
Tg3A
Sm7
Hd3
Cg3_7J
Cg3_7I
Cg3_7H
Cg3_7G
Cg3_7F
Cg3_7E
Mg3_7
Cg3_7D
Cg3_7C
Tg3_7
Cg3_7A
Cg3_7
Ch3_7
Cg3_7B
Cg3_7K
Cg3_7L
Cg3B
Cg3B-like
Cg3_7-likeH
Cg3_7-likeG
Cg3_7-like
Cg3_7-likeD
Cg3_7-likeC
Cg3_7-likeB
Cg3_7-likeA
Cg3_7-likeE
Cg3_7-likeF
Aj6
Hl6
Mm6
Hs6
Dr6
On6
Aj1
DmSTRICA
DmDAMM
DmDECAY
Lm1
Sf1
DmDCP1
DmDRICE
Es7
Pm3
Pme3
Es37
Pj1

```
Mg37_1       112 -TNCPELHLKPKVFIFQACRGSDL-GSG-V--ERKVIRNRDPDQADAMASYVE---------------------------------------------------------
Mg37_2       112 -TNCPELILKPKVFIFQACRGGGL-GRG-V--NRKVIR--DPNQADAGAEYDA---------------------------------------------------------
Mg37_4       112 -ENCPQLILKPKIFIFQCSRKPDS-RQG-E--HITVQDTLKNKV------------------------------------------------------------------
Cg3C-like_A  111 -NKIPAFTGKPKAFFIQSCRGEAY-QET-M--VQPDNDE-----------------------------------------------------------------------
Cg3C-like_B  110 -NKIPAFTGKPKAFFIQSCRGEAH-QDT-M--LQPDYDE-----------------------------------------------------------------------
Ca3C         110 -DKLKYFAGKPKCFFIQACRGSSP-QGV-V-DLPDDKE------------------------------------------------------------------------
Cg3C         110 -DKLKYFAGKPKCFFIQACRGSSP-QGV-V-DLSDDKE------------------------------------------------------------------------
Aj3          130 -WSAPKLVGKPKVFVIQACRGIKS-ARG-ILDRSDCNPP-----------------------------------------------------------------------
Bl3_7        111 -DSLKCMAGSPKMFIIQSCRGKQK-QPG-V-DRADEEPGDEG--------------------------------------------------------------------
Av3          113 ----SNLVGKPKVFIFQACQGHEY-MDG-K--DATDAP------------------------------------------------------------------------
Ep3          113 ----TNLVGKPKIFIFQACQGHEY-MDG-V--DATDAP------------------------------------------------------------------------
Dr7          111 -DVCKSLVGKPKLFFIQACRGSEF-DDG-V--QTDSGPPNDT--------------------------------------------------------------------
Ss7          108 -DMCKSLVGKPKLFFIQACRGSEF-DDG-I--QTDSGPPNDT--------------------------------------------------------------------
Xl7          111 -DNCRSLVGKPKLFFIQACRGHEF-DDG-L--EADSGSVNDS--------------------------------------------------------------------
Hs7          111 -DRCKTLLEKPKLFFIQACRGTEL-DDG-I--QADSGPIND---------------------------------------------------------------------
Mm7          111 -DRCKTLLEKPKLFFIQACRGTEL-DDG-I--QADSGPIND---------------------------------------------------------------------
Co7          111 -DKCKSLVGKPKLFFIQACRGSEF-DEG-I--QTDSGPANDT--------------------------------------------------------------------
Meg7         111 -DKCKSLIGKPKLFFIQACRGSEF-DEG-I--QTDSGPANDT--------------------------------------------------------------------
On3          111 -DRCKSLVGKPQLFFIQACRGTDL-DPG-I--ETDSET------------------------------------------------------------------------
Dr3          111 -DRCPSLVGKPKLFFIQACRGTEL-DPG-V--ETDHTDHPD---------------------------------------------------------------------
Xl3          111 -DRCKTLVGKPKIFFIQACRGTEL-DSG-I--ETDSCSEPR---------------------------------------------------------------------
Hs3          111 -DRCRSLTGKPKLFIIQACRGTEL-DCG-I--ETDSGVDDD---------------------------------------------------------------------
Mm3          111 -DYCRSLTGKPKLFIIQACRGTEL-DCG-I--ETDSGTDEE---------------------------------------------------------------------
Bf37         111 -DRCPGLVGKPKLFFLQACRGNEF-DHG-V--DMPDALPEVQ--------------------------------------------------------------------
Cg3A         111 -DKCPMLVGKPKLFFLQSCRCPNL-EQS-V--E--DSVSYKSFLSED---------------------------------------------------------------
Tg3A         111 -DRCPTLVGKPKLFFLQTCRASRI-DRV-L--DLTDSVSDRSELSEEL--------------------------------------------------------------
Sm7          112 -DQCLDLRGKPKLFFIQACRGMAL-DDG-V--FVCDGPLSNSK-------------------------------------------------------------------
Hd3          107 -DRCPALAGKPKLFFIQACRGKQF-DDG-A--DALSVKNDGPEDDEDE--------------------------------------------------------------
Cg3_7J       121 -GKCKALKNKPKLFFIQACRIPET-KST-Q-DHYKAGVGFDSGISTHNKKGGQDQRTTRDKTDFEGFSGDTETDSDRESD-VSDTDVEEDESSLSDIEPTATGHGDGKEP
Cg3_7I       121 -RKCKALKNKPKLFFIQACRIPET-KST-H-DQYDAGVGFDSGISTHNKKGGQDQRTTRDKTDFGGSSGGTETDSDEESD-VSDTDVEEDESSLSDSDIEPTAKGQGVCY
Cg3_7H       122 -TNCKALKNKPKLFFIQACRIPHT-KAT-K-TERIKGIGFDSGVHLPSRGDEHDQRTAGDLTDFDGSTCETDTDSDSESDDVSDTDVEEDESSLFDIEITARGHEEREKI
Cg3_7G       121 -KECTGLIDKAKIFFIQACRIPES-KST-T-AHKSLGIGLDPGVPYNPPQ------------------------------------------------------------
Cg3_7F       131 -TNCPGLRNKPKIFFIQACRIPDS-KAT-A-DQWKAEIGLEKGCSPPKAAKYN---------------------------------------------------------
Cg3_7E       132 -SRCKALQGKPKLFFVQACRSRSN-QGP---SRYSKGVNVVEPISKNCSNPRQGCDQTESLSKDSAPQSTEVVDGSVDQVKRRRYTLDQSDNAHDSNPKSASDTATTDES
Mg3_7        126 -DYCEGLKDKPKFFFIQACRTSNT-TVL-G--MDSGHILTITERKQPPGGVDDDEEMHVDSKITDNVDANYISTNKDNLDTRHMSTADT---------------------
Cg3_7D       127 -QHCNALRGKPRLFFVQACRGNDV-DKG-V-YLNENTENF----------------------------------------------------------------------
Cg3_7C       126 -RNCRHLKGKPKMFFIQACRSRLD-VQR-A-DEVDMGVEVHLVETKATTSAGSPARFFTRGDMAPTSSDNTEDMAPARPVTTGDVVGNYDDYSGRRPEEFRRNFTAATLD
Tg3_7        117 GKLFRSLKGKPKLFFIQACRA----IFG-K--SNEESIDPGISVEVDFGPPFNNNQ------------------------------------------------------
Cg3_7A       140 -LSC------PCTFFIQACRSRFG-VINHV--NYDKGVAVLLKSN-------------SEDALKDIPLTDTVDAIPSKIKISKEDEEKRIRDITASNKMTEFQKYMAWLK
Cg3_7        145 -RSCPALTEKPCTFFIQACRSRFG-VENHV--NYDEGAILSVNSTSENMTKERQSETDSEDASKGDPLKDTSDAKRSIIKRSKEDEEERVREITARYKIDQFGEYMTWFR
Ch3_7        133 -MNCRALKGKPKIFFIQACRGRTN-ESQNVLDFVDRGVQISVESEPSSTLHPSTQFSRSSYNNPVMQCGLDETDSVPDAKAIRAKDIRSGHKIFVESS------------
Cg3_7B       133 -MNCRALKGKPKIFFIQACRSRTS-DSQNILDCVDTGVQISVQSDPSSEPHPSTQHSRSSYNNPIMQCGLDETDSVPDAKAVRPENIRSGHKIFVESS------------
Cg3_7K       135 ----ETLNKIPKIFFIQACRGKEL-DIG-H--PLDGNKNVGQDQN-----------------------------------------------------------------
Cg3_7L       147 ----RKLKPIPKIFFIQACRAEEN-STT-D--VDTGIRVGYRQENEFQYQ------------------------------------------------------------
Cg3B         111 -NKCLSLAGKPKIFFIQACRGTKF-DDG-V--DMNVADAKGFMDV-----------------------------------------------------------------
Cg3B-like    111 -NECLSLAGKPKIFFIQAC----H-DDMNV--GDGKEV------------------------------------------------------------------------
Cg3_7-likeH  138 -NNCRNLVLKPKIFLVQTCDAYLK-HED-K--SQFSGRA-----------------------------------------------------------------------
Cg3_7-likeG  142 -NNCRNLALKPKIFLVQTCNAQLA-QKD-R--SLFMD-------------------------------------------------------------------------
Cg3_7-like   128 -DKCPSLAMKPKLFFMQLCPAFTYNIAD-C--VKDTGSSS----------------------------------------------------------------------
Cg3_7-likeD  130 -DKCKGLLLKPKIFMLQLEEALRD-SKD-G--IET---------------------------------------------------------------------------
Cg3_7-likeC  132 -DKCRDLLLKPKIFMLQLERALGD-YIE-S--PAT---------------------------------------------------------------------------
Cg3_7-likeB  148 -DKCKKLLLKPKIFMLQLEGTRLD-SMD-T--VHQT--------------------------------------------------------------------------
Cg3_7-likeA  158 -DKCKKLLLKPKIFMLQFEKVECG-SKE-G--GQPGRQ------------------------------------------------------------------------
Cg3_7-likeE  125 -NKCRGLLLKPKIFMLQLEEAPGD-KKD-G--TKA---------------------------------------------------------------------------
Cg3_7-likeF  127 -DKCRDLLLKPKIFMLQLEKVPES-SAE-S--VSPQS-------------------------------------------------------------------------
Aj6          111 -EKCPSLVGKPKVFFIQACRGEQH-EVG-V--IEADSGDQTDS-------------------------------------------------------------------
Hl6          111 -EYCPSLVGKPKIFLVQACRGEQH-EIG-V--LEADSLDQTDS-------------------------------------------------------------------
Mm6          111 -DKCQSLVGKPKIFIIQACRGSQH-DVP-V--VPLDMVDHQTD-------------------------------------------------------------------
Hs6          110 -DKCHSLVGKPKIFIIQACRGNQH-DVP-V--IPLDVVDNQTE-------------------------------------------------------------------
Dr6          111 -DKCRSLVGKPKIFIWQACRGDKH-DDP-V--TPMDVVDSQVT-------------------------------------------------------------------
On6          111 -DKCQNLVGKPKIFIIQACRGKEF-DVS-V--TVTPASTMESE-------------------------------------------------------------------
Aj1          126 -NNCSTLIGKPKIFIIQACRGGTA-EFS-A--MIGSAAT-----------------------------------------------------------------------
DmSTRICA     107 -LRNRTLKDKPKLIFVQACKGDCQ-LGG-F--MT----------------------------------------------------------------------------
DmDAMM       111 -FRNPTLSGKPKILIVQACKGPLR-ADA-K--K-----------------------------------------------------------------------------
DmDECAY      108 -DNCKTLKNKPKLFFIQACRGANL-EKA-V--EFSSFAVMTRELVPE---------------------------------------------------------------
Lm1          110 -DRCPSLAGKPKLFFIQACQGDKL-DGG-I--TLR---TETD--------------------------------------------------------------------
Sf1          110 -DKCPTLAGKPKLFFIQACQGDRL-DGG-I--TLSR--TETD--------------------------------------------------------------------
DmDCP1       110 -TFCPSLAGKPKLFFIQACQGDRL-DGG-I--TLEKGVTETD--------------------------------------------------------------------
DmDRICE      110 -NHCPSLAGKPKLFFIQACQGDRL-DGG-V--TMQRSQTETD--------------------------------------------------------------------
Es7          114 -DKCPSLAGKPKLFFIQACRGMRT-DKG-V--RMKH-PKGLG--------------------------------------------------------------------
Pm3          116 -ERCAGLAGKPKLYFIQACRGPDV-DKG-V--NMTRAVRGMA--------------------------------------------------------------------
Pme3         116 -ERCASLAGKPKLYFIQACRGLDV-DKG-V--NMSRAVRGMA--------------------------------------------------------------------
Es37         112 -DQCKSLAGKPKIFFIQACRGEGL-DSG-V--TLAHSRPSDE--------------------------------------------------------------------
Pj1          112 -DQCPTLAGKPKLFFIQACRGEGL-DAG-T--TLVHQKTRDE--------------------------------------------------------------------
```

```
Mg37_1         160 --------------------------------------------------------------------------------------------------------------
Mg37_2         158 --------------------------------------------------------------------------------------------------------------
Mg37_4         151 --------------------------------------------------------------------------------------------------------------
Cg3C-like_A    145 --------------------------------------------------------------------------------------------------------------
Cg3C-like_B    144 --------------------------------------------------------------------------------------------------------------
Ca3C           144 --------------------------------------------------------------------------------------------------------------
Cg3C           144 --------------------------------------------------------------------------------------------------------------
Aj3            166 --------------------------------------------------------------------------------------------------------------
Bl3_7          149 --------------------------------------------------------------------------------------------------------------
Av3            143 --------------------------------------------------------------------------------------------------------------
Ep3            143 --------------------------------------------------------------------------------------------------------------
Dr7            148 --------------------------------------------------------------------------------------------------------------
Ss7            145 --------------------------------------------------------------------------------------------------------------
Xl7            148 --------------------------------------------------------------------------------------------------------------
Hs7            147 --------------------------------------------------------------------------------------------------------------
Mm7            147 --------------------------------------------------------------------------------------------------------------
Co7            148 --------------------------------------------------------------------------------------------------------------
Meg7           148 --------------------------------------------------------------------------------------------------------------
On3            144 --------------------------------------------------------------------------------------------------------------
Dr3            147 --------------------------------------------------------------------------------------------------------------
Xl3            147 --------------------------------------------------------------------------------------------------------------
Hs3            147 --------------------------------------------------------------------------------------------------------------
Mm3            147 --------------------------------------------------------------------------------------------------------------
Bf37           148 --------------------------------------------------------------------------------------------------------------
Cg3A           151 --------------------------------------------------------------------------------------------------------------
Tg3A           154 --------------------------------------------------------------------------------------------------------------
Sm7            150 --------------------------------------------------------------------------------------------------------------
Hd3            150 --------------------------------------------------------------------------------------------------------------
Cg3_7J         226 WQNLEKRQTEILKKLKKMEKGQTEILKKLKRVENGQTKILKRQKRMENKVWHLGREYMAAANQNKEPNALAAANQNEEEPSPQATANQ------------------NEEE
Cg3_7I         226 WQKLEKRQTEILKKLKKMEKGQTEILKKLKRVENGQTKILKRQKRMENKVWHLGREYMVAANQNKESNALAAANQNEEEPSPQTTTNQ-------------------NEE
Cg3_7H         228 WQNLEKRQAEIFKKLKKME----------------------------NQVWLLGREYIVAAKENRKPEKKGPRSPIVGNSQTAAYENKKPSVHDNEELNSQNAASENDEE
Cg3_7G         167 ------------------------------------------------------------------------------------------PSPTQPNPAQPNPAQTNPAQ
Cg3_7F         180 --------------------------------------------------------------------------------------------------------------
Cg3_7E         237 EHNFEEDAYRGNEASQHTELTNELMDWSSSDDVIPCLDVDIIRDENKDLVEEPSFIEIGKNWVRKGF-------------------------------------------
Mg3_7          210 --------------------------------------------------------------------------------------------------------------
Cg3_7D         163 --------------------------------------------------------------------------------------------------------------
Cg3_7C         232 PLYKNYQVHGLSRQYPGYYHVHAQASLQRQEPARTGT-------------------------------------------------------------------------
Tg3_7          166 --------------------------------------------------------------------------------------------------------------
Cg3_7A         227 SIDATSTASKDDSKEVLQLREALKCDVFKPQETEDIMVLCKGYEKYTLDFFY----------------------------------------------------------
Cg3_7          251 NTNAPSLASEDDSEAVQKLREALNCDVLKPQETEEIMVLCKGYEKYTLDFFY----------------------------------------------------------
Ch3_7          229 --------------------------------------------------------------------------------------------------------------
Cg3_7B         229 --------------------------------------------------------------------------------------------------------------
Cg3_7K         172 --------------------------------------------------------------------------------------------------------------
Cg3_7L         189 --------------------------------------------------------------------------------------------------------------
Cg3B           151 --------------------------------------------------------------------------------------------------------------
Cg3B-like      141 --------------------------------------------------------------------------------------------------------------
Cg3_7-likeH    172 --------------------------------------------------------------------------------------------------------------
Cg3_7-likeG    174 --------------------------------------------------------------------------------------------------------------
Cg3_7-like     164 --------------------------------------------------------------------------------------------------------------
Cg3_7-likeD    160 --------------------------------------------------------------------------------------------------------------
Cg3_7-likeC    162 --------------------------------------------------------------------------------------------------------------
Cg3_7-likeB    179 --------------------------------------------------------------------------------------------------------------
Cg3_7-likeA    191 --------------------------------------------------------------------------------------------------------------
Cg3_7-likeE    155 --------------------------------------------------------------------------------------------------------------
Cg3_7-likeF    159 --------------------------------------------------------------------------------------------------------------
Aj6            149 --------------------------------------------------------------------------------------------------------------
Hl6            149 --------------------------------------------------------------------------------------------------------------
Mm6            149 --------------------------------------------------------------------------------------------------------------
Hs6            148 --------------------------------------------------------------------------------------------------------------
Dr6            149 --------------------------------------------------------------------------------------------------------------
On6            149 --------------------------------------------------------------------------------------------------------------
Aj1            160 --------------------------------------------------------------------------------------------------------------
DmSTRICA       136 --------------------------------------------------------------------------------------------------------------
DmDAMM         139 --------------------------------------------------------------------------------------------------------------
DmDECAY        150 --------------------------------------------------------------------------------------------------------------
Lm1            144 --------------------------------------------------------------------------------------------------------------
Sf1            145 --------------------------------------------------------------------------------------------------------------
DmDCP1         147 --------------------------------------------------------------------------------------------------------------
DmDRICE        147 --------------------------------------------------------------------------------------------------------------
Es7            150 --------------------------------------------------------------------------------------------------------------
Pm3            153 --------------------------------------------------------------------------------------------------------------
Pme3           153 --------------------------------------------------------------------------------------------------------------
Es37           149 --------------------------------------------------------------------------------------------------------------
Pj1            149 --------------------------------------------------------------------------------------------------------------
```

```
Mg37_1       160 ------------------------------------------------------------------------------------------------------YDEEFGEI
Mg37_2       158 ------------------------------------------------------------------------------------------------------YDEIYGDT
Mg37_4       151 ------------------------------------------------------------------------------------------------------TIPDAVDT
Cg3C-like_A  145 -------------------------------------------------------------------------------------------------------EEDDAEK
Cg3C-like_B  144 -------------------------------------------------------------------------------------------------------EEEDAEK
Ca3C         144 ----------------------------------------------------------------------------------------------------EVMIDQVVGK
Cg3C         144 ----------------------------------------------------------------------------------------------------EVMIDQVVGK
Aj3          166 ----------------------------------------------------------------------------------------------------AQVIPTPPTQ
Bl3_7        149 ----------------------------------------------------------------------------------------------------QLDVSDVMSS
Av3          143 ---------------------------------------------------------------------------------------------------------PPDKR
Ep3          143 ---------------------------------------------------------------------------------------------------------PADKR
Dr7          148 ------------------------------------------------------------------------------------------------------IETDANPR
Ss7          145 ------------------------------------------------------------------------------------------------------LETDANPR
Xl7          148 ------------------------------------------------------------------------------------------------------LETDANPR
Hs7          147 --------------------------------------------------------------------------------------------------------TDANPR
Mm7          147 --------------------------------------------------------------------------------------------------------IDANPR
Co7          148 ------------------------------------------------------------------------------------------------------LETDANPR
Meg7         148 ------------------------------------------------------------------------------------------------------LETDANPR
On3          144 ------------------------------------------------------------------------------------------------------------DG
Dr3          147 ---------------------------------------------------------------------------------------------------------IPDGR
Xl3          147 -----------------------------------------------------------------------------------------------------------EEI
Hs3          147 -----------------------------------------------------------------------------------------------------------MAC
Mm3          147 -----------------------------------------------------------------------------------------------------------MAC
Bf37         148 ------------------------------------------------------------------------------------------------------DELDAGNK
Cg3A         151 ------------------------------------------------------------------------------------------------------SERSFSGM
Tg3A         154 ------------------------------------------------------------------------------------------------------DQGGVPGI
Sm7          150 ------------------------------------------------------------------------------------------------------SADAATTI
Hd3          150 -----------------------------------------------------------------------------------------------------MDVKDETVT
Cg3_7J       318 PSPQTTASQNEEEPSPQTTANQNVEPSPQTTANQNKESSLQASGNEDSKSHSAADENKNASGVTGDIEDDID-AKRRRLY--ESRGSGDDIDPPRGQRIYP-PAPAPVEF
Cg3_7I       317 PSPRTTANQN-KEPSPQTTANQNKEPCLQAGGNG------------DSNSHSAADENENASGVTADLEDDIDPAKRRRLYPLESGGSGDDIDPARGRKIYP---PAPLEF
Cg3_7H       310 PCPHDSADEKPNPQ----------------------------------------------------------------YSPEVNADHEDDIDPAKGQKVYFTNTSAPVEF
Cg3_7G       187 PNPAQPNPAQTNSAQTNPAQPNPAQPNPAQTNSAQPNPAQTNIAHPNPVKTN----PVKPNPV-------IQTDLDTTNIKDSSVEDQHEIDAPKPNIIYP---PAHLEV
Cg3_7F       180 ----------------------------------------------PAEHNPVKPNPVKPAPQTTIEMEADSYLPDATKTKDPSVEDKDKIDPPKAHITNP---PAPVEI
Cg3_7E       304 ---------------------------------------------------------------------------------------------FKRRNLFARPVQDVMTT
Mg3_7        210 ---------------------------------------------------------------------------------------------------YLSRRQLVHQV
Cg3_7D       163 ----------------------------------------------------------------------------------------------ESNKDQSEDISLNQDV
Cg3_7C       269 ---------------------------------------------------------------------------------------------YKFKPTEPEPESEPIEV
Tg3_7        166 --------------------------------------------------------------------------------LNSQPEKPCLEQDGDELNPGMVSFSKEIKP
Cg3_7A       279 --------------------------------------------------------------------------------------------------------PEPTIS
Cg3_7        303 --------------------------------------------------------------------------------------------------------PEPSIS
Ch3_7        229 -------------------------------------------------------------------------------------------------ERLRASMEEEIVL
Cg3_7B       229 -------------------------------------------------------------------------------------------------ERLRASVEEEIVL
Cg3_7K       172 -------------------------------------------------------------------------------------------------------DDEPNRA
Cg3_7L       189 -------------------------------------------------------------------------------------------------------DDPLNRS
Cg3B         151 --------------------------------------------------------------------------------------------------------EPQFSL
Cg3B-like    141 --------------------------------------------------------------------------------------------------------EPQFSR
Cg3_7-likeH  172 -------------------------------------------------------------------------------------------------------TIPPSSL
Cg3_7-likeG  174 -------------------------------------------------------------------------------------------------------GISPPPL
Cg3_7-like   164 -------------------------------------------------------------------------------------------------------SRLQPEI
Cg3_7-likeD  160 -------------------------------------------------------------------------------------------------------GTQAPKP
Cg3_7-likeC  162 -------------------------------------------------------------------------------------------------------GTQAHKP
Cg3_7-likeB  179 -------------------------------------------------------------------------------------------------------GTQAPKP
Cg3_7-likeA  191 -------------------------------------------------------------------------------------------------------GR-QAPS
Cg3_7-likeE  155 -------------------------------------------------------------------------------------------------------ETEAPQP
Cg3_7-likeF  159 -------------------------------------------------------------------------------------------------------DTHTEKP
Aj6          149 --------------------------------------------------------------------------------------------------RGSVTTISQGSI
Hl6          149 --------------------------------------------------------------------------------------------------RGSITTVSEGLP
Mm6          149 -------------------------------------------------------------------------------------------------KLDNVTQVDAASV
Hs6          148 ------------------------------------------------------------------------------------------------KLDTNITEVDAASV
Dr6          149 ----------------------------------------------------------------------------------------------------NDMVVDAGVL
On6          149 ----------------------------------------------------------------------------------------------------EKVFMDASTI
Aj1          160 -------------------------------------------------------------------------------------------------DYEEPLETSVGVK
DmSTRICA     136 -------------------------------------------------------------------------------------------------------------D
DmDAMM       139 -------------------------------------------------------------------------------------------------------------M
DmDECAY      150 -----------------------------------------------------------------------------------------------------PAAAVQPIT
Lm1          144 --------------------------------------------------------------------------------------------------------GAP-LS
Sf1          145 --------------------------------------------------------------------------------------------------------GSPSTS
DmDCP1       147 --------------------------------------------------------------------------------------------------------GESSTS
DmDRICE      147 --------------------------------------------------------------------------------------------------------GDSSMS
Es7          150 ---------------------------------------------------------------------------------------------------VQTDSYSKPDN
Pm3          153 ---------------------------------------------------------------------------------------------------VQT-DSI--EE
Pme3         153 ---------------------------------------------------------------------------------------------------VQTDNIGSTEE
Es37         149 -------------------------------------------------------------------------------------------------------IDSGVIA
Pj1          149 -------------------------------------------------------------------------------------------------------IDSGYQA
```

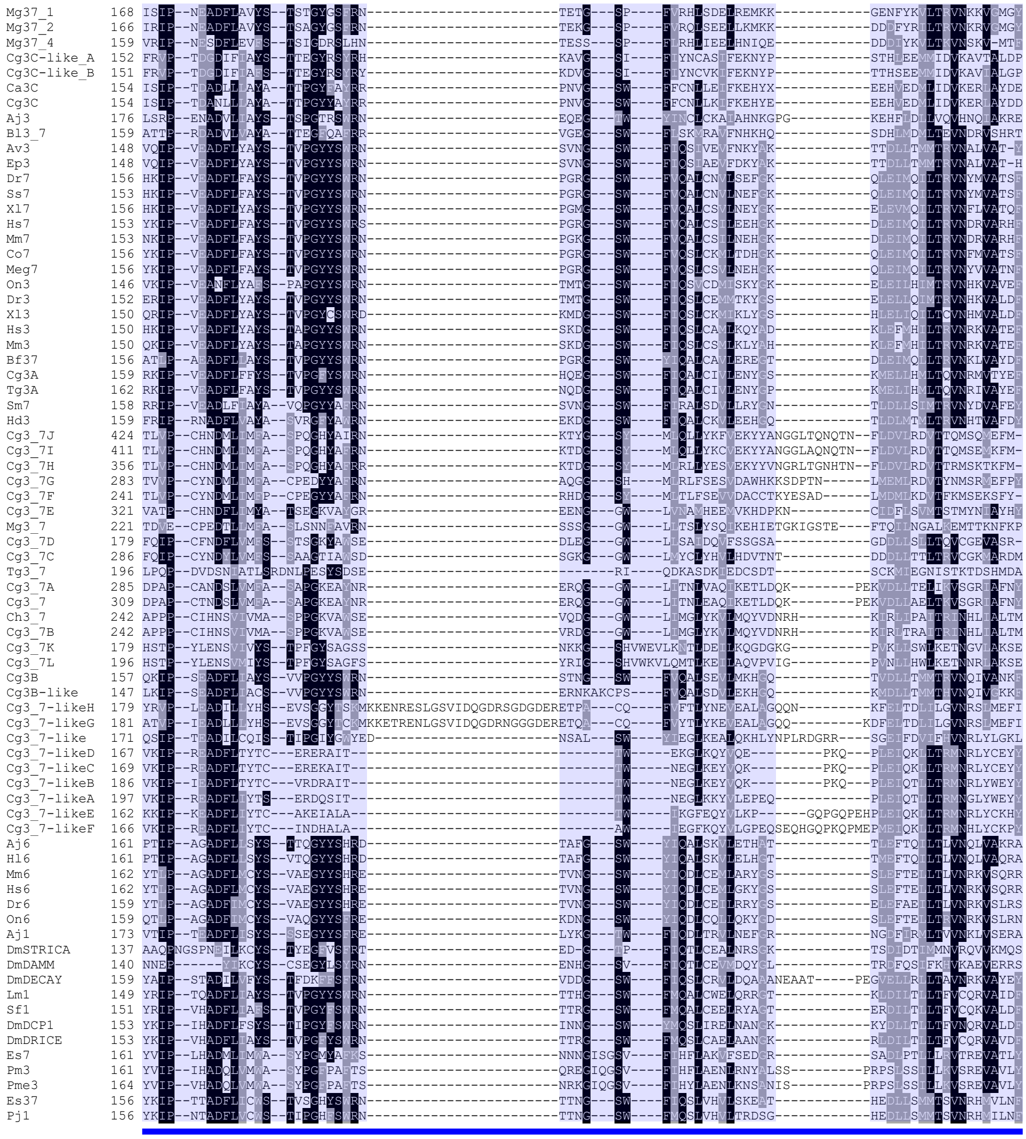

```
Mg37_1        231 KPKHP-------SSRIVTQMPCFISHLT-KDLVFNQKVRKCIHTIN------------------------------
Mg37_2        229 KPNFP-------NSGDVTQMPCFISHLT-KELYLKQMN--------------------------------------
Mg37_4        221 EPGHT-------ASKDMRQMPYFVSHLT-KDLYLKQTN--------------------------------------
Cg3C-like_A   215 RWQRTK------DKKEIAQMPCSWSTLT-KRFYFIAATL-------------------------------------
Cg3C-like_B   214 SWRQTK------DKKEIAQMPCSWSTLT-KRFYFIAAAL-------------------------------------
Ca3C          217 EWQ---------TNEGFKQMPCTWSTLT-KRFFLAPQQLAE-----------------------------------
Cg3C          217 EWQ---------TNEGFKQMPCTWSTLT-KRFFLAPQQLAE-----------------------------------
Aj3           241 TQG---------NDVAVKQMPCLVTSFT-KKFLL------------------------------------------
Bl3_7         222 AI----------GSKPAKQMPCQVSTFT-KKFYF------------------------------------------
Av3           210 QSRTNDP-----YSDRKKQIPSIVSMLR-KDLYFFPNNLVDS----------------------------------
Ep3           210 QSRTNDP-----YSDRKKQIPSIVSMMR-KDFYFYPENITNGNN--------------------------------
Dr7           219 ESWSEDP-----RFSEKKQIPCVVSMLT-KELYFN-----------------------------------------
Ss7           216 ESWSEDP-----RFSEKKQIPCMVSMLS-KELYFGP----------------------------------------
Xl7           219 ESYSDDP-----QFSKKKQIPCVVSMLT-KELYFKSC---------------------------------------
Hs7           216 ESQSDDP-----HFHEKKQIPCVVSMLT-KELYFSQ----------------------------------------
Mm7           216 ESQSDDP-----RFNEKKQIPCMVSMLT-KELYFSR----------------------------------------
Co7           219 ESWSDDP-----RFSEKKQIPCVVSMLT-KELYF------------------------------------------
Meg7          219 ESQSDDP-----RFSEKKQIPCVVSMLT-KELYF------------------------------------------
On3           209 ESASNSP-----GFDAKKQIPCIVSMLT-KEMYFSR----------------------------------------
Dr3           215 ESTSNMP-----GFDAKKQIPCIVSMLT-KEMYFTP----------------------------------------
Xl3           213 ET-----------FHAKKQIPCVVSMLT-KSFYFFK----------------------------------------
Hs3           213 ESFSFDA-----TFHAKKQIPCIVSMLT-KELYFYH----------------------------------------
Mm3           213 ESFSLDS-----TFHAKKQIPCIVSMLT-KELYFYH----------------------------------------
Bf37          219 HSNSDNP-----YMNRKKQIPCIMSMLT-KDLFLSPKSQ-------------------------------------
Cg3A          222 ESCSDEH-----FTDEVKQMPCIVSMLT-RYVYFRPKKPDIRD---------------------------------
Tg3A          225 ESCSDEE-----FTERSKQMPCIVSLLT-RYVYFRPKKPDIRD---------------------------------
Sm7           221 ESTAANP-----SFCGKKQMPSFVSTLT-KHVIFPPKKPRNPSKLM------------------------------
Hd3           222 SSKG--------KIKEKKQIPCITSMLT-KDVFFRKT---------------------------------------
Cg3_7J        496 ------------GSKEYTIVPCVVHKLR-KDVIFTQGKNMGSTKFTVKLSEIISTCGMSFFTWSDKN---------
Cg3_7I        483 ------------GSKEYTIVPCIVHKLR-KDVIFTQGKSMGSTKFTFKLSEIISTLGMSFFTWSNKN---------
Cg3_7H        428 ------------GSMEYTIVSCVVHKLR-KDVIFTHGTNMSRTKFTIKPCDLE-----------------------
Cg3_7G        352 ------------GLKKYKIVPNIVHKLK-KDVIFNGGNIMYT----------------------------------
Cg3_7F        309 ------------GPKEYKTVPSVVHKLR-KDVIFKAKFSSRLAF--------------------------------
Cg3_7E        386 ETNTGEP-----STSGFKTAPTLQHKLS-HDLIFRNK---------------------------------------
Mg3_7         292 SDTTS-------RLYGAFSPGCFTHCLS-RDVLFLEKQYR------------------------------------
Cg3_7D        241 EAFIPNNE----RAHCSKSTACVYHKLS-KDIYFPSSKTKYSDVDC------------------------------
Cg3_7C        350 ETYCPSTP----HYDKAKSAACVYHRLS-KDIYLQPVSHL------------------------------------
Tg3_7         257 KYKFDSDTNT--YRVVEDSGPTTQHSIIYNRLGNSPVNQQTGGFQHSP----------------------------
Cg3_7A        352 ETETRSL-----TDSGHKSVPCLYHRLS-EDIIIWPEEMKKAKQLA-EEECSEWKKFSDWDPNKDEQARIRVKDEV
Cg3_7         376 ETETGSL-----TDSGHKSVPCLYHKLS-KDIIIWPEEMKKTMQLLVEKEGSEWEKFSFWDP--------------
Ch3_7         308 QTVTNDP-----SLNFMKCMPVMSHTLE-RDIVFVPRNIQ------------------------------------
Cg3_7B        308 HTITSDP-----SLNFMKCMPVMSHTLE-RDIVLVPKNIQ------------------------------------
Cg3_7K        248 RDI---------NDGTYKPLLSICHTLT-REILFTR----------------------------------------
Cg3_7L        265 EYEI--------DDIEYKPLLSICHTLT-RNIIFDTNNN-------------------------------------
Cg3B          220 QSNTSHS-----DMNEKKQIPCVTSMLT-KEVYFTSK---------------------------------------
Cg3B-like     213 QPDTSHP-----DMNEKKQIPLVTSMLT-KEVYFTIK---------------------------------------
Cg3_7-likeH   269 DKEHYRSDEYNKPWNIEPKIPICVDQLT-KRLVLGTNTG-------------------------------------
Cg3_7-likeG   271 DKEHYRSDEDNKPWTIEPMIPICVDQLT-KRLVLRTNLG-------------------------------------
Cg3_7-like    243 EEQGIRP--------SQYSVPHTTSTLT-KSLHFLPKNRTM-----------------------------------
Cg3_7-likeD   222 S-----------TQGMTVDTPCVTSLLT-REAYLKIEEPSPK----------------------------------
Cg3_7-likeC   224 S-----------KKHMTVDAPCVTSLLT-REAYLKKEEPSPK----------------------------------
Cg3_7-likeB   241 S-----------NEGMRVDTPCVTSLLT-REAYLKTEEPSTK----------------------------------
Cg3_7-likeA   252 MN----------EEQMRMDTPCVTSLLT-REAYLKK----------------------------------------
Cg3_7-likeE   223 S-----------DEKMTVDTPCVTSLLT-REAYLKIEEPSPK----------------------------------
Cg3_7-likeF   233 I-----------EAGSYVDIPCVTSLLT-REAYLKLEEPSPK----------------------------------
Aj6           224 VERCLDP-----RMIGKKQIPCFVSMLT-KRLIFTPKKK-------------------------------------
Hl6           224 VERCLDS-----RMIGKKQIPCFVSMLT-KRLIFRPKVR-------------------------------------
Mm6           225 VDFCKDP-----DAIGKKQVPCFASMLT-KKLHFCPKPSK------------------------------------
Hs6           225 VDFCKDP-----SAIGKKQVPCFASMLT-KKLHFFP----KSN---------------------------------
Dr6           222 VLNCKDR-----SAVGKKQVPCFASMLT-KKLFFRPKK--------------------------------------
On6           222 IPTCS-P-----KFIGKKQMPCFASMLT-KKLYFRPKK--------------------------------------
Aj1           236 IQGSGKP-----ELEGKKQMPCFLSKLT-RQLHLTAQT--------------------------------------
DmSTRICA      201 K---------------DRQIPSVTSTLT-SKYVFGDYI--------------------------------------
DmDAMM        199 TMTG------------SKQVPSEESHNF-DKPFYFGNYAKNT----------------------------------
DmDECAY       229 QSNTKNE-----ALNQMKEMPNFMSTLT-KTFQLRVKPKT------------------------------------
Lm1           212 ESNTPNND----VMHRQKQIPCITTMLT-RLLRFEEK---------------------------------------
Sf1           214 ESNAPDSA----MMHQQKQVPCITSMLT-RLLVFGKKQSH------------------------------------
DmDCP1        216 ESNVPATP----MMDRQKQIPCLTSMLT-RILRFGDKPNGNKAG--------------------------------
DmDRICE       216 ESCTPDTP----EMHQQKQIPCITTMLT-RILRFSDKQLAPAGRV-------------------------------
Es7           227 ESYHPDNK----DLDQNKQIPYIVSTLM-RKVYFPVKT--------------------------------------
Pm3           230 ESDIDSQN----QYHKNKQVPYIHSTLL-REIY-------------------------------------------
Pme3          233 ESDIGSRN----KYHENKQVPYIHSTLL-REIYF------------------------------------------
Es37          219 ESNTPSQQ----HMHGKKQSASIVSTLM-RKVFLSPKY--------------------------------------
Pj1           219 ESNCPSQH----HMHGKKQAACIVTTLM-RKVYFTQKY--------------------------------------
```
