# Supplementary material for: Phylogenetic analysis of the caspase family in bivalves: implications for programmed cell death, immune response and development
Source: BMC Genomics. 2021 Jan 25;22:80. doi: 10.1186/s12864-021-07380-0 (PMC7836458; doi:10.1186/s12864-021-07380-0)
Supplement: Supplementary file 6 — Additional file 6:. Alignment of CARD domains of initiator caspases. [file 12864_2021_7380_MOESM6_ESM.pdf]

Additional File 6: Alignment of CARD domains of initiator caspases.

```
Dr9_CARD         1 MEQKHRQILQRHRLNLVKAL-NPE-DVCDKLLSKG---VFTQDMIDE--------IQS--KATR-RDQARQLVKDLETRGSLAFPAFLDSLRETGHNDLAELLQSG
Hs9_CARD         1 MDEADRRLLRRCRLRLVEEL-QVD-QLWDALLSRE---LFRPHMIED--------IQRAGSGSR-RDQARQLIIDLETRGSQALPLFISCLEDTGQDMLASFLRTN
Xl9_CARD         1 MEQELRDILRRNRVRLVQSL-QVK-ELWDLLVERG---VFSNDMIEE--------IQR--EGTR-RDQARKLLVELETRGSQAFPLFLLCLKETAQHDLADFLQSD
Cg2-likeA_CARD   1 MDQIHKDALRRTRVALVKDL-DVN-NVCDELLSQD---IFTQLMMEY--------IMS--ERTR-IDKVRRLLDDLVRRGPDAYLKFLDVLKKSGYPFLADQIVKN
Cg2-likeB_CARD   1 MDQIHRDALRRTRVALVKDL-DVN-IVCDELLSQD---IFTPLMIKH--------IMV--EGTR-NDKVCRLLNTLVEIGTNAYQAFLNVLKTTGYEFLANQIVQN
Cg2-likeC_CARD   1 MDDRERSILRRSRSLFIRDLVDVV-TVCDRLYQRE---VLSEGMKNE--------ILS--ETTR-ENQIRTLMDIIPKRGQKAYGAFYDVLEETYQYNLASILYPG
Cg2A_CARD        1 MEDADRRVLQVNRTFLVKNTANPD-DVANELFSNE---IFTEGMKDE--------VEV--EKLK-EKKVRKLLDILPRRGPVAFQIFIKVLQDTNNDHVALKLKSG
Cg2B_CARD        1 NEDEWKNSIRRNYSYFKNNIADSL-DVAEYLFGETNPPIITDNQKEQ--------IKA--ENTT-SKKVGKMLDILMKRGPRVPQCLFDAFMETHNEECAKKLAPY
Cg2C_CARD        1 NEEEWKDSIRRNYSFFKDNIADPH-DVVQYLFGQTNPPIITHDYLDE--------IKA--ERTP-SKQVEKMLDILLIRGPMVPQCLFDAFVETHNEECAKKLAPY
Mg2-like_CARD    1 MEQKHRNILQTNFAYLIKNLHNVE-EICDHLMSGD---ILTSGMMDS--------IKH--KKPRPTGQTRELLSILPRRGMKAYKSFIHALHETGNGEVGDHLEKN
DmDRONC_CARD     1 MPKRHREHIRKNLNILVEWT-NYE-RLAMECVQQG---ILTVQMLRNTQDLNGKPFNMDEKDVR-VEQHRRLLLKITQRGPTAYNLLINALRNINCLDAAVLLESV
Aj2_CARD         1 MEELHKEAIRANYVSLSQEM-DLS-LIVPYLIQAN---IYKPYMVES--------FKA--KDGK-LAQNQAFLNSIETRGPNAYQAFINALQSVGQGHLAEAIQNE
Hd8_CARD         1 RHNSHRDTLRKCYHHIISDLELVNSSVLDRLLEDD---VMSVSDKDS--------IIM--KGTK-KSQNEEFYRYIYGK---------------------------
Dr2_CARD         1 MTKWERLALRRNSVKMLQDL-VVD-DLLIQCLQQDG--ILTDSMAES--------IMA--KPTS-QGRSHQLLFLLPKRGPRAFSTFCSALKETEQHHLCKLLMDF
Xl2_CARD         1 MQQHHRKALQRLRVSLASEM-IIE-ELLDHLVSSE---ILTNNMHSN--------IMA--YRSD-YAQNVALLNLLPKRGPRAFSAFCNALHSTNQEHLAQQVEKE
Hs2_CARD         1 MHPHHQETLKKNRVVLAKQL-LLS-ELLEHLLEKD---IITLEMREL--------IQA--KVGS-FSQNVELLNLLPKRGPQAFDAFCEALRETKQGHLEDMLLTT
CeCED3_CARD      1 MRQDRRSLLERNIMMFSSHL-KVD-EILEVLIAKQ---VLNSDNGDM--------INS--CGTV-REKRREIVKAVQRRGDVAFDAFYDALRSTGHEGLAEVLEPL
Cg2_CARD         1 MDKAKKNILIKNRVELVENINIED-GLLTELLSRQ---VLTQRTAKS--------IQA--KKTT-FDQTEELLDKI-------------ALKSNGFEALSEALIAN
Ca2_CARD         1 MDKAKKNILIKNRVELVENINIED-GLLTELLSRQ---VLTQRTAKS--------IQA--KKTT-FDQTEELLDKI-------------ALKSNGFEALSEALIAN
Xl1_CARD         1 MT-AQLNKVRKAIINGCNPA-MIS-DLLDDLQDKH---VLKDYEVEH--------INK--KNNTSRDRCRDMIDSVKKKGEYSSNILLESLVKNHKTLAESLGLH-
Dl1_CARD         1 MADKELFRVRSKFVEKVSKD-LIK-QLLDDIIADC---IVNDGERDS--------IIE--ENVNRADRARDLLDTVKRKGDVASRKFIAHIQRRDLTLYSELGLAE
Hs1_CARD         1 MADKVLKEKRKLFIRSMGEG-TIN-GLLDELLQTR---VLNKEEMEK--------VKR--ENATVMDKTRALIDSVIPKGAQACQICITYICEEDSYLAGTLGLSA
Mm1_CARD         1 MADKILRAKRKQFINSVSIG-TIN-GLLDELLEKR---VLNQEEMDK--------IKL--ANITAMDKARDLCDHVSKKGPQASQIFITYICNEDCYLAGILELQS
Hs5_CARD         1 VKKDNHKKKTVKMLEYLGKD-VLH-GVFNYLAKHD---VLTLKEEEK--------KKY--YDTKIEDKALILVDSL-RKNRVAHQMFTQTLLNMDQKITSVKPLLQ
Hs4_CARD         1 MAEGNHRKKPLKVLESLGKD-FLT-GVLDNLVEQN---VLNWKEEEK--------KKY--YDAKTEDKVRVMADSMQEKQRMAGQMLLQTFFNIDQISPNKKAHPN
```
