# Supplementary material for: Phylogenetic analysis of the caspase family in bivalves: implications for programmed cell death, immune response and development
Source: BMC Genomics. 2021 Jan 25;22:80. doi: 10.1186/s12864-021-07380-0 (PMC7836458; doi:10.1186/s12864-021-07380-0)
Supplement: Supplementary file 7 — Additional file 7:. Alignment of DED-DED domains of initiator caspases. [file 12864_2021_7380_MOESM7_ESM.pdf]

Additional File 7: Alignment of DED-DED domains of initiator caspases. Green underscored: DED domains.

```
Dr10_CARD          1 MCFQRALLRVQESLSADELQDLLFLCSDLLCSRDLNTVSSGRQLFTLLQNQDLL--SLEDPSLLIELLSITKRTRLMRSLQSDACMQTSGTADQPLQQQQR---------
Hs10_CARD          1 VSFREKLLIIDSNLGVQDVENLKFLCIGLVPNKKLEKSSSASDVFEHLLAEDLL--SEEDPFFLAELLYIIRQKKLLQ-HLNCTKEEVERLLP----TRQR---------
Xl10_CARD          1 MDFNSMLLRIDDGLGREDIEALKFLCRDVLRKNKLLSVQSGHELFQQLMTEDLI--NEDNYFLLGELLYIINHHSLLH-DLGTNKVEVQKALP----HHWK---------
Mm8_CARDm          1 MDFQSCLYAIAEELGSEDLAALKFLCLDYIPHKKQETIEDAQKLFLRLREKGML--EEGNLSFLKELLFHISRWDLLVNFLDCNREEMVRELRDP--DNVQ---------
Hs8_CARD           1 MDFSRNLYDIGEQLDSEDLASLKFLSLDYIPQRKQEPIKDALMLFQRLQEKRML--EESNLSFLKELLFRINRLDLLITYLNTRKEEMERELQTP--GRAQ---------
Xl8_CARD           1 ADMNKLLFEISEDLDKTETLAMIFLCEKRVTAQEKENIKDAKTLFLCLKKKDLI--CYNDLSFLKELLYRIGRNDLLRGKLGVRTEEIKRIIE----VSPQ---------
Dr8_CARD           1 -------------------------------------------------------------------------------------------------------------
Hdd8_CARD          1 HSFHAMLLKIDSDLHQADLQALKFLIKDNLPKNKLTRMSTGHELFTALETSKLI--DTSNRWLLVEMFNKIGRLDLID----YVDEEITEDLKSTYLPTGK---------
Aj8_CARD           1 -------------------------------------------------------------------------------------------------------------
Mg8-like_CARD      1 -------------------------------------------------------------------------------------------------------------
Tt8_CARD           1 LDYRKLLIDVHENLVSRDLAALKFLCSDIMPVAVLERVTSGLELLDLLETRRYI-VAADNVLFLAELLYYIGRKDLLR-KLNVTDNEVRTSLTDK--QHSH---------
Cg8B_CARD          1 YDYGEFLGCVDSGLDTSDVDNLKFLLRGVLPDSKIQRAERGLDLFTELQNAGVIDIDNADLELLEECLYRIHRKDLLK-KMARDTNLVEQRLKRLQADLQKKKISYMNSA
Mc8B_CARD          1 TDLTKGLITIDGELESDDIKSLKFLCQDHIAQKTLEKIEDGIQIFNELERLGKIDTSRGEVDYLLESLCRCHRIDLIR-KMGFNQIQVKNRVES---GRSN---------
Mco8A_CARD         1 CLTKDILVDICSDLDSDDMDNLKFLMKDVAGLHKLMPATIALDLFSAIENNKDV--ALLNGRFLAECFELMGRTDLVR-RIGLDPTKVEKEMGQ---TIYS---------
Mc8A_CARD          1 CLTKDILVDICSDLDSDDMDNLKFLMKDVAGLHKLMPATIALDLFSAIENNKTV--ALLNGRFLAECFELMGRTDLVR-RIGLNPTQVEKEMGQ---TNYS---------
Mg8A_CARD          1 CLTKDILVDICSDLDSDDMDNLKFLMKDVAGLHKLMPATNALDLFSAIENNTHV--AFHNGRFLAECFELMGRTDLVG-RLGLNPTQIEKEMGQ---HNYS---------
Ch8A_CARD          1 GDFRAILLDIDAHLDSKDLELLKFLCKDVVSFSVLDKCRRCLDVFANLEQKGKL--KEQDSKYLQECFHYMQRKDLIR-KLSGNPQQLENRIRAD--GPNY---------
Cg8A_CARD          1 GDFKAILLDIDAHLDSKDLELLKFLCKDVVSFSVLGKCQRCLDVFANLEQKGKL--KEENSKYLQECFHYMQRKDLIR-KLSGDPQQLENRIRAN--GPNY---------
Bf8_CARD           1 FNFRKALKDIDDDLTSGDIAALKFLCRDFIPAAKLENAKGSLDVFRQLENKGLL--GQNNLMFLTELLYRIQRADLLR-KLAWRQEQVSGEIGR---GRAL---------
Bl8_CARD           1 FNFRRALKDIDDDLTSGDIAALKFLCRDFIPAAKLENAKGSLDVFRQLENKGLL--GSNNLMFLTELLHRIQRADLLR-KLAWHPDQVSGEIER---GRAL---------
Cg8-likeC_CARD     1 -------------------------------------------------------------------------------------------------------------
Mt8_CARD           1 SDYRQIMNTVMKNMRGENLKLSLYLCQDIIPQSI--DFKTCLQFVQHLEQSNKL--SYINISLLVEILYRINRIDLLK-KFNISKIHLEDNYLSM--GHTH---------
Hl8_CARD           1 -------------------------------------------------------------------------------------------------------------
Cg8-likeB_CARD     1 -------------------------------------------------------------------------------------------------------------
Cg8-likeA_CARD     1 -------------------------------------------------------------------------------------------------------------

Dr10_CARD        100 --ISRYRQFLYELSESICDQDLKNIKFLLMKT--LSRKKLEQNLTLLQLFLEMEKEDLLGENNLDVLQRIFADI--------YPALGRKINQ
Hs10_CARD         95 --VSLFRNLLYELSEGIDSENLKDMIFLLKDS--LPKTEM----TSLSFLAFLEKQGKIDEDNLTCLEDLCKTV--------VPKLLRNIEK
Xl10_CARD         95 --ISPYRQMLYELSENVTGEDEKRILFILP----LHK-KHKENKTFLDVLCQLEKENAITEDNVKLLEEVFRKV--------SPDLLKIIEK
Mm8_CARDm         98 --ISPYRVMLFKLSEEVSELELRSFKFLLNNE--IPKCKLEDDLSLLEIFVEMEKRTMLAENNLETLKSICDQV--------NKSLLGKIED
Hs8_CARD          98 --ISAYRVMLYQISEEVSRSELRSFKFLLQEE--ISKCKLDDDMNLLDIFIEMEKRVILGEGKLDILKRVCAQI--------NKSLLKIIND
Xl8_CARD          96 --ISPYRILLYDISQGLSKKEVEDLKYLLD----LSTAKTENA-SILEIFLELEKVGKLHPDDLQKLKHDLETI-------GCKNLSRNIED
Dr8_CARD           1 ---SAYRKMLLKISEDMTEENFRAAKFLLD----LPRAKLGRSTSFLDALIEMEKQQRLGPDNLDELYRILEKC-----DKQLAVMIERFRN
Hdd8_CARD         96 --ISDYRLMLFDTAQDLTGEDTNKLSFLLDK---IPKAKRGKVSSGLDLVTLMEESKIISPASVDRLRQNLVAL-------TRQDLVRKLDQ
Aj8_CARD           1 ---TNYRTVLADVADELTDDDAKALAFELV----IPRGERGKIINGRTLIDVMEKNEMVSEDKVDQLLKLLRKR-------RYNVAATKLEG
Mg8-like_CARD      1 -HAYLTTQLSNYIDSSIDKDQYEQLLNRLKSVL-TPKDRNNAKTIMHILESMEKKSFIAPGKYADLKKILSEFD-----ERIARDVIEPIEE
Tt8_CARD          98 --VKQHRVLLHTICQELTTKELLSLKFQCSAK--ITRAKLETIKTPQELILELERQRVITHDNFTYLLSLLRDI-------HRQDLVLKVSQ
Cg8B_CARD        110 KFLSPFRVLLFEIGEDIDDEEFKSVMFTLTSV--VPKGQLSKMKSIFDLFLFLEKKDEMSHTKVDVLLKVLKVI-------DRADLIPKVNK
Mc8B_CARD         98 --FSPFRLLLFEIAEEMETADLKTALFTLSSV--IPRSKHDKIKSICDVFIQLERSGDLSPVNVEVLYKLLKMM-------DRGDLITKVRA
Mco8A_CARD        96 --VRPFRILLYKISEDLASDDVELLASFCLRVFKLTRQEKETIKSAFDLFVLLEKRGIIEVAHTNPLKDMVKSL------ENRGDLVDLVNE
Mc8A_CARD         96 --VRPFRILLYKISEDLASDGVELLASFSLRVFKLTRQEKESIKSAFDLFVILEKRGIIEVAHTNPLKEMVKSL------ENRGDLVDLVDE
Mg8A_CARD         96 --VRPFRILLYKISEDLASDDVELLASFSLRVFELTQQEKATIKSALDLFVLLEKRGIIEIDDTNPLKEMVKSL------ENRGDLVDLVDK
Ch8A_CARD         97 --LTEFRILLFDIAEDCGKDELEKMKFYSRTTLHLPKKMVDKVRSCLDLFNVLEKEEKITVIDVKTLEQLIKTT-------GDEELLEFLYQ
Cg8A_CARD         97 --LTEFRILLFDIAEDCGKDELEKMKFYSRTTLHLPKKMVDKVKSCLDLFNVLEKEEKITNIDVNTLEQLIKTT-------GDEELLESVYQ
Bf8_CARD          96 --LNPFRVMLLQVSEELTSRDFDDMKFMAAAY--ISRNKLETVDNFLSLAIALEQGMHLATDNVQVLHELLQTQ-------ERPDLKNIIQN
Bl8_CARD          96 --LNPFRVMLLRVSEELTNRDFDDMKFMAAAY--ISRNKLEMVDNFLSLVIALEQGLHLTSDNVQVLHELLQTQ-------ERPDLKDMIRN
Cg8-likeC_CARD     1 ---SERRKTLYSVGEEIGQDELKKLKALLTVELRVGKRTMDTVKDVWDCLDIMEKRLLDSELFSFLKTVYTTDE-------RLLEIIDDFAK
Mt8_CARD          95 --FTPFRILMFELTDELSQTDFDCLKFQFSDH--MKQRQLDETTDVVSLLIKLEELGVVDCDDFRKMIEILELI-------VNPRPFHKF--
Hl8_CARD           1 ---------MYSIAEQIPSSDLKKMKDFCLDQELLEPYQLEDSQNFLDFFMLLEDEAVIEADNIGLLKDFTKLL----NIHKVKELVDKYEE
Cg8-likeB_CARD     1 ---SNCRMLLIEINEKLSSDDFQSLTFLAKD--IVNRKKIKEKENRLEFFDDLEKKAVIKCGTEETDLLFLKQAFLIMGKNNLVSALDEVGE
Cg8-likeA_CARD     1 ---SNCRMLLIEINEKLSSDDFQSLTFLAKD--IVNRKRIKENENRLEFFEDLEKKAVIKCGTEETDLLFLKQAFLIMGKNNLVSALDKVGE
```
