# Supplementary material for: Phylogenetic analysis of the caspase family in bivalves: implications for programmed cell death, immune response and development
Source: BMC Genomics. 2021 Jan 25;22:80. doi: 10.1186/s12864-021-07380-0 (PMC7836458; doi:10.1186/s12864-021-07380-0)
Supplement: Supplementary file 8 — Additional file 8: Primers for quantitative gene expression analysis of caspases in Crassostrea gigas. [file 12864_2021_7380_MOESM8_ESM.pdf]

Additional File 8: Primers for quantitative gene expression analysis of caspases in *Crassostrea gigas*. EF1: elongation factor 1, RS18: ribosomal protein S18, RL7: ribosomal protein L7, Fwd: forward primer; Rev: reverse primer.

***C. gigas* caspase (GenBank ID)**	**Primer**	**Sequence**	**Size amplicon (bp)**	**Annealing temperature (°C)**	**Final primer conc.**	**Final MgCl2 conc.**	**Efficiency**
Cg3B	Cg3B_Fwd	5'-GGCCGACTTCCTGATTGCTT-3'					
(AEB54802 [27]) EKC34324 [29]) AVH80607 [28])	Cg3B_Rev	5'-GTCGGAGTGGGAGGTGTTAG-3'	196	60	0.3 mM	2.5 mM	1.96
Cg3A	Cg3A_Fwd	5'-ATCTCGCCAAAGTCTCGCTG -3'					
(XP_011445226)	Cg3A_Rev	5'-CTTTACCGGTGGACGGATGG -3'	191	62	0.2 mM	2.5 mM	2.05
Cg3C	Cg3C_Fwd	5'-ATGCCATGTTGAGACCGACC -3'					
(EKC43168 [30])	Cg3C_Rev	5'-CGTACGAATGCCATCCTCGT -3'	151	62	0.3 mM	2.5 mM	2.04
Cg3/7	Cg3/7_Fwd	5'-TGGTACACCAAACTCCACACC -3'					
(AMZ04158 [33])	Cg3/7_Rev	5'-CTGGCTCTTGGGTATCTGATGT -3'	164	60	0.3 mM	4 mM	1.94
Cg2A	Cg2A_Fwd	5'-GCCAACCCTGATGATGTTGC -3'					
(AEB54803 [27])	Cg2A_Rev	5'-CTGGAATGCTACGGGACCAC -3'	150	60	0.3 mM	2.5 mM	2.04
Cg8B	Cg8B_Fwd	5'-GTCCGTGGTGCCTAAAGGA -3'					
(AKP95634 [21])	Cg8B_Rev	5'-CTCTTTGGCTTGCAGGGAAAT -3'	184	60	0.3 mM	2.5 mM	2.11
CgEF1	CgEF1_Fwd	5'-CGGAGATGCTGGTATGGTCC-3'					
(AB122066)	CgEF1_Rev	5'-GTGCAGCCTTGGTGACTTTG-3'	178	60	0.3 mM	2.5 mM	2.01
CgRS18	CgRS18_Fwd	5'-CAGCCAGGTCATGTCCAACA-3'					
(AB199895)	CgRS18_Rev	5'-TTGGCCACACCAACAGTTCT-3'	165	60	0.3 mM	4 mM	1.94
CgRL7	CgRL7_Fwd	5'-ACAAGCCAGGAAGAATGGCA-3'					
(AJ557884)	CgRL7_Rev	5'-TGCAATGAAGGGTTCAGCGA-3'	204	60	0.3 mM	2.5 mM	1.99
